# CtpB Facilitates *Mycobacterium tuberculosis* Growth in Copper-Limited Niches

**DOI:** 10.3390/ijms23105713

**Published:** 2022-05-20

**Authors:** Oliver Shey-Njila, Ahmed F. Hikal, Tuhina Gupta, Kaori Sakamoto, Hind Yahyaoui Azami, Wendy T. Watford, Frederick D. Quinn, Russell K. Karls

**Affiliations:** 1 Department of Infectious Diseases, College of Veterinary Medicine, University of Georgia, Athens, GA 30602, USA; onjila@gmail.com (O.S.-N.); hikal@uga.edu (A.F.H.); tgupta@uga.edu (T.G.); hind.yahyaouiazami@uga.edu (H.Y.A.); watfordw@uga.edu (W.T.W.); fquinn@uga.edu (F.D.Q.); 2 Department of Bacteriology, Immunology and Mycology, College of Veterinary Medicine, Benha University, Toukh 13736, Egypt; 3 Department of Pathology, College of Veterinary Medicine, University of Georgia, Athens, GA 30602, USA; kaoris@uga.edu

**Keywords:** tuberculosis, CtpB, copper, transport, mycobacterium, evolution, respiration, ATPase, virulence, nutritional immunity

## Abstract

Copper is required for aerobic respiration by *Mycobacterium tuberculosis* and its human host, but this essential element is toxic in abundance. Copper nutritional immunity refers to host processes that modulate levels of free copper to alternately starve and intoxicate invading microbes. Bacteria engulfed by macrophages are initially contained within copper-limited phagosomes, which fuse with ATP7A vesicles that pump in toxic levels of copper. In this report, we examine how CtpB, a P-type ATPase in *M. tuberculosis*, aids in response to nutritional immunity. In vitro, the induced expression of *ctpB* in copper-replete medium inhibited mycobacterial growth, while deletion of the gene impaired growth only in copper-starved medium and within copper-limited host cells, suggesting a role for CtpB in copper acquisition or export to the copper-dependent respiration supercomplex. Unexpectedly, the absence of *ctpB* resulted in hypervirulence in the DBA/2 mouse infection model. As *ctpB* null strains exhibit diminished growth only in copper-starved conditions, reduced copper transport may have enabled the mutant to acquire a “Goldilocks” amount of the metal during transit through copper-intoxicating environments within this model system. This work reveals CtpB as a component of the *M. tuberculosis* toolkit to counter host nutritional immunity and underscores the importance of elucidating copper-uptake mechanisms in pathogenic mycobacteria.

## 1. Introduction

*Mycobacterium tuberculosis*, the primary cause of human tuberculosis (TB) remains a leading cause of morbidity and mortality in humans. The World Health Organization estimated that 10 million people developed TB disease in 2019 resulting in 1.2 million deaths [1]. Fueling these active infections is a reservoir of latent infections of approximately 23% of the world population [2]. This global burden of disease and the rise in multidrug-resistant TB emphasize the need to characterize novel processes in the pathogenesis of *M. tuberculosis* as potential targets for treatment strategies and antimicrobial design [1].

For replication, *M. tuberculosis* must acquire trace amounts of copper and other essential metals from its host. Copper functions in single-electron transfer reactions in enzymes including the cytochrome *aa3-bc1* respiration supercomplex of *M. tuberculosis* [3]. The gene *Rv2043* encodes cytochrome *aa3* subunit 1, which contains the copper-B catalytic site. Multiple saturating transposon mutagenesis screens indicate that *Rv2043* is essential [4,5,6]; thus, copper is essential for the replication of this pathogen. Due to its high reactivity, copper is universally toxic in excess in both prokaryotic and eukaryotic cells, necessitating levels to be tightly controlled. Among the mechanisms underlying its toxic effects is the disruption of protein function through the formation of thiolate bonds with iron–sulfur centers [7]. The sequestration and release of copper and other essential trace elements in different niches within host organisms to protect against pathogens has been termed nutritional immunity [8]. The level of free copper ions in unstressed eukaryotic cells is estimated to be extremely low, below 10^−18^ M [9]. In contrast, this metal is concentrated within phagosomes of macrophages stimulated with pro-inflammatory molecules to form an antimicrobial environment. The stimulation of RAW 264.7 murine macrophages by lipopolysaccharide or IFNγ was shown to upregulate the copper importer CTR and increase trafficking of the copper transporter ATP7A to post-Golgi vesicles; these vesicles fuse with phagosomal compartments resulting in the ATP7A- and copper-dependent killing of *E. coli* mutants that are unable to produce the copper-exporting P-type ATPase CopA [10]. Additionally, the infection of murine C57BL/6 peritoneal macrophages with *M. tuberculosis* bacilli increased copper levels to as high as 0.4 mM within phagosomes [11]. Mutants of *M. tuberculosis* unable to synthesize the cytoplasmic copper-storage metallothionein MymT, the cytoplasmic-membrane copper-efflux P-type ATPase CtpV, or the outer-membrane protein MctB were more sensitive to macrophage killing (reviewed in [12]). Regulators of copper-toxicity-response genes in *M. tuberculosis* include the copper-sensing transcriptional repressors CsoR and RicR [13,14,15].

How *M. tuberculosis* bacteria acquire copper inside the host is less clear, but components have begun to be identified. One regulator of copper acquisition is transcription sigma factor SigC. The short-term artificial induction of *sigC* in *M. tuberculosis* strain Erdman elevated the transcription of genes *Rv0096-Rv0101* and nearby *Rv0103*/*ctpB*, while prolonged *sigC* induction further increased the expression of the same genes but also elevated copper-toxicity-response genes: *ricR*, *mymT*, and *Rv0846–Rv0850* [16]. The deletion of *sigC* impaired replication in copper-starved medium and attenuated virulence in SCID mice, supporting SigC as an important regulator of copper acquisition and pathogenesis [16]. The genes *Rv0097-Rv0101* contribute to the synthesis of isonitrile lipopeptides and aid in zinc acquisition [17,18]. In silico analysis supports CtpB as a transmembrane P-type ATPase with a copper/silver binding motif [19]. It was recently reported that the constitutive heterologous expression of CtpB from *M. tuberculosis* strain H37Rv in fast-growing, non-pathogenic *M. smegmatis* resulted in elevated ATPase activity in the CtpB-enriched plasma membrane when incubated with copper (I) ions and a tolerance of high levels (2–2.5 mM) of copper (II) ions [20]. The same study reported that deleting *ctpB* in avirulent *M. tuberculosis* strain H37Ra did not alter growth phenotypes in Sauton medium supplemented with CuSO_4_ (5 to 100 µM) or intracellular copper levels in the presence of 50 µM CuSO_4_, which led to a conclusion that CtpB functions in copper transport, but possibly not in copper detoxification.

To elucidate the functions of CtpB, we sought to examine its potential roles in copper acquisition and virulence. Our studies reveal that the controlled induction of *M. tuberculosis ctpB* in *M. smegmatis* increases sensitivity to copper, while *ctpB*-deletion mutants of *M. tuberculosis* and *Mycobacterium bovis* Bacille Calmette-Guérin (BCG) develop growth defects only after serial subculture in medium lacking copper, suggesting that internal stores of the metal must be depleted for phenotypes to manifest. Surprisingly, deleting *M. tuberculosis ctpB* enhanced replication and virulence in a murine infection model, but reduced replication in adipocytes cultured without copper supplementation. Homologs of CtpB are only conserved in intracellular pathogenic mycobacteria, supporting the evolution of this protein to counter copper nutritional-immunity mechanisms.

## 2. Results

### 2.1. Expressing M. tuberculosis ctpB in M. smegmatis Negatively Impacts Growth in Copper-Supplemented Medium

**Figure 1:**
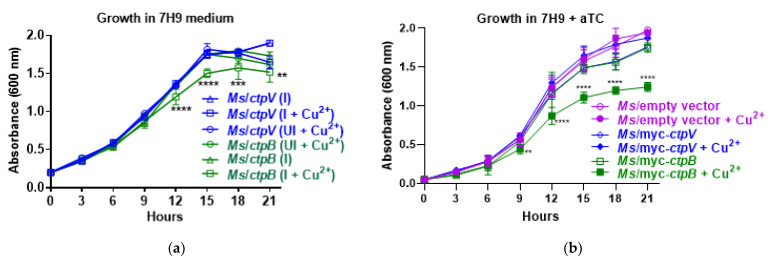
Heterologous expression of *M. tuberculosis ctpB* in *M. smegmatis* negatively impacts growth in copper-supplemented medium. (**a**) Strains of *M. smegmatis* encoding N-terminal *myc*-tagged *M. tuberculosis ctpB* or *ctpV* controlled from an aTC-inducible promoter (*Ms*/*ctpB* and *Ms*/*ctpV*, respectively) were cultured in Middlebrook 7H9 medium and subcultured in fresh medium without (UI) or with (I) inducer aTC (2 µg/mL) and/or 0.1 mL CuCl_2_ (Cu^2+^) and growth was monitored by measuring absorbances at 600 nm. (**b**) Growth of strains of *M. smegmatis* expressing *ctpB* or *ctpV* without myc tags or the empty vector plasmid (vector) were subcultured in 7H9 medium with aTC (2 µg/mL) +/−0.1 mM CuCl_2_. Averages of three biological replicates with standard error are shown. Significant differences based on ANOVA with multiple comparisons for the indicated groups versus all others are shown (** *p* < 0.01, *** *p* < 0.001, **** *p* < 0.0001).

To begin to assess whether *M. tuberculosis* CtpB plays a role in copper import, an anhydrotetracycline (aTC)-controlled expression system was utilized to study growth impacts following the expression of *ctpB* or *ctpV*, encoding a known *M. tuberculosis* copper-exporting P-type ATPase, in *M. smegmatis*. Each gene was cloned into a plasmid downstream of the aTC-inducible mycobacterial promoter, P*_myctetO_* [21]. The growth of *M. smegmatis* transformants was evaluated in Middlebrook 7H9 medium containing 6 µM copper sulfate with additional copper associated with bovine-serum-albumin supplementation. No differences were observed between strains induced for *ctpB* or *ctpV* with 2 µg/mL aTC; however, if the level of CuCl_2_ in the growth medium was increased to 0.1 mM, then the growth of the *ctpB* strain, but not the *ctpV* strain, was significantly reduced (Figure 1a). The growth of either strain without aTC induction but with 0.1 mM CuCl_2_ addition was not different from strains that were only induced with aTC, indicating that both the induction of *ctpB* and the addition of excess copper was necessary for the growth defect to manifest (Figure 1a). Plasmids encoding CtpB or CtpV with N-terminal myc tags were also examined in the presence of aTC. Only the induction of myc-*ctpB* in the presence of 0.1 mM copper ions significantly reduced growth (Figure 1b). No differences were detected in the absence or presence of added copper between strains carrying an empty vector or one encoding tagged *ctpV* (Figure 1b). Together, these observations indicate that inducing the expression of *M. tuberculosis ctpB* negatively impacts growth in a copper-rich environment, and the presence of an N-terminal myc tag does not prevent CtpB function. The data are consistent with CtpB importing, rather than exporting, copper ions.

### 2.2. CtpB Expression Does Not Reverse the Growth Defect of a M. tuberculosis SigC Null Mutant in Copper-Chelated Medium

**Figure 2:**
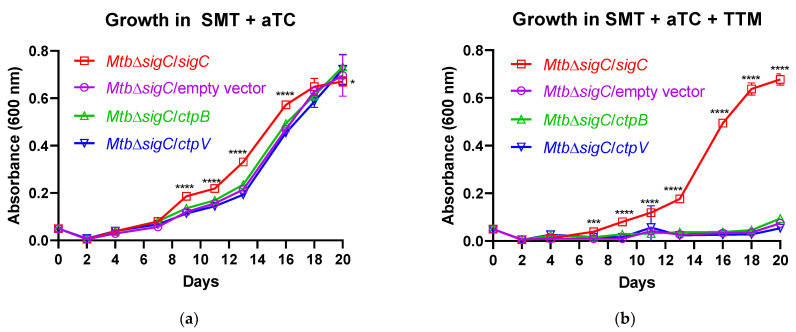
The growth defect of a *M. tuberculosis sigC* mutant in copper-chelated medium is not reversed by *ctpB*. Growth of derivatives of *M. tuberculosis* Δ*sigC* that were transformed with an empty vector plasmid or with one encoding the indicated *M. tuberculosis* gene encoding SigC, CtpB, or CtpV with an N-terminal myc tag was examined after passaging three times in SMT prior to inoculation into fresh medium containing 50 ng/mL aTC (inducer) alone (**a**) or with 50 ng/mL aTC and 0.01 mM TTM (copper chelator) (**b**). Shown are the averages with standard error of three experiments performed in triplicate. Significant differences between the indicated strain relative to all others are indicated (* *p* < 0.5, *** *p* < 0.001, **** *p* < 0.0001).

If CtpB functions as a copper-import pump, then the expression of *ctpB* should be elevated in response to copper restriction. To test this hypothesis, *ctpB* transcription was examined by RT-qPCR of *M. tuberculosis* strain Erdman subcultured in SMT with and without the addition of the copper (II) chelator tetrathiomolybdate (TTM). The results normalized to housekeeping gene *sigA* reveal that *ctpB* was induced eight-fold in the presence of TTM (Figure S1A). The response to copper limitation is mediated in part through SigC, since this sigma factor was shown to direct *ctpB* transcription in vitro [22] and after artificial *sigC* induction in *M. tuberculosis* cultures [16]. Moreover, *sigC* is required for *M. tuberculosis* growth in copper-starved medium [16]. Therefore, we next asked whether artificial *ctpB* induction could compensate for the impaired growth of a *M. tuberculosis sigC*-deletion mutant under copper-limiting conditions. To test this, we compared the growth of Δ*sigC* derivatives harboring plasmids that differed in terms of the gene fused to a myc tag located downstream of an aTC-inducible promoter; the variations were *ctpB*, *ctpV*, *sigC*, or no gene. After initial growth in 7H9 medium, strains were passaged three times in SMT to reduce copper loads prior to subculture in SMT with the inducer (50 ng/mL aTC) alone or with the inducer and copper chelator (10 µM TTM). Without TTM addition, the Δ*sigC* mutant strains containing a plasmid expressing CtpB, CtpV, or a vector alone grew nearly as well as the *sigC*-complemented strain; however, growth was absent for all strains except for the *sigC*-complemented strain in the presence of the copper chelator (Figure 2). As CtpB is localized to the cytoplasmic membrane, restored growth following the induction of *sigC*, but not *ctpB*, is consistent with SigC also controlling a system for copper import across the outer membrane. This system likely involves the *Rv0096-Rv0101* operon, which requires *sigC* for expression under copper-starved conditions [16]. Thus, these data indicate that the transcription of *ctpB* in *M. tuberculosis* is elevated in response to copper depletion, but *ctpB* alone cannot compensate for the loss of *sigC* in response to copper starvation.

### 2.3. Development of a Growth Defect in M. bovis BCG and M. tuberculosis CtpB Mutants in Copper-Deficient Medium

**Figure 3:**
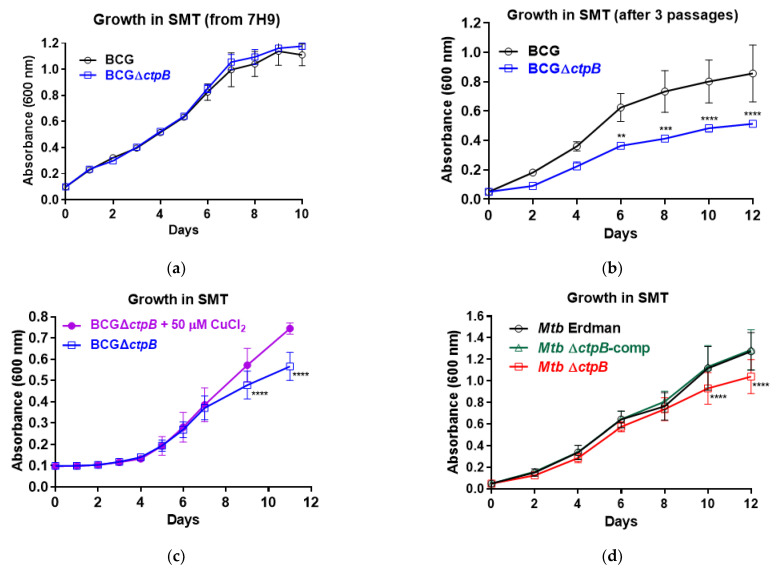
Growth of BCG and *M. tuberculosis ctpB* mutants is impacted by copper availability. (**a**) Growth of *M. bovis* BCG or *ctpB*-deletion mutant (BCGΔ*ctpB*) after subculture from 7H9 to SMT medium. (**b**) Growth of BCG and BCGΔ*ctpB* after three serial passages in SMT. (**c**) Growth of BCGΔ*ctpB* in SMT +/− 50 μM CuCl_2_ after three passages in SMT. (**d**) Growth of *M. tuberculosis ctpB*-deletion mutant (*Mtb*Δ*ctpB*), parent strain (*Mtb* Erdman), and complemented mutant (*Mtb*Δ*ctpB*-comp) in SMT medium after three passages in SMT medium. Results shown are the averages of a minimum of three biological replicates in triplicate (**a**,**c**,**d**) or three biological replicates (**b**). Significant growth differences for the indicated strains and conditions are shown (** *p* < 0.01, *** *p* < 0.001, **** *p* ≤ 0.0001).

To confirm the CtpB function in copper uptake, a *ctpB*-deletion mutant was first generated in *M. bovis* BCG (Figure S2). Following initial subculture of the mutant from 7H9 to SMT media, no differences in growth were observed (Figure 3a). However, after three passages in SMT, growth of the mutant significantly lagged behind its parent (Figure 3b). The addition of 50 µM copper chloride partially reversed the growth defect (Figure 3c). The *ctpB* gene was also deleted and confirmed in the *M. tuberculosis* strain Erdman (Figure S2). After three passages in SMT, a significant growth defect was evident in the mutant relative to the parent Erdman or a *ctpB*-complemented mutant (Figure 3d). Together, these data support that *ctpB* is required for the optimal growth of *M. tuberculosis* and *M. bovis* BCG under copper-restricted conditions.

### 2.4. A Replication Defect Manifests in Adipocytes Infected with M. tuberculosis ΔCtpB

**Figure 4:**
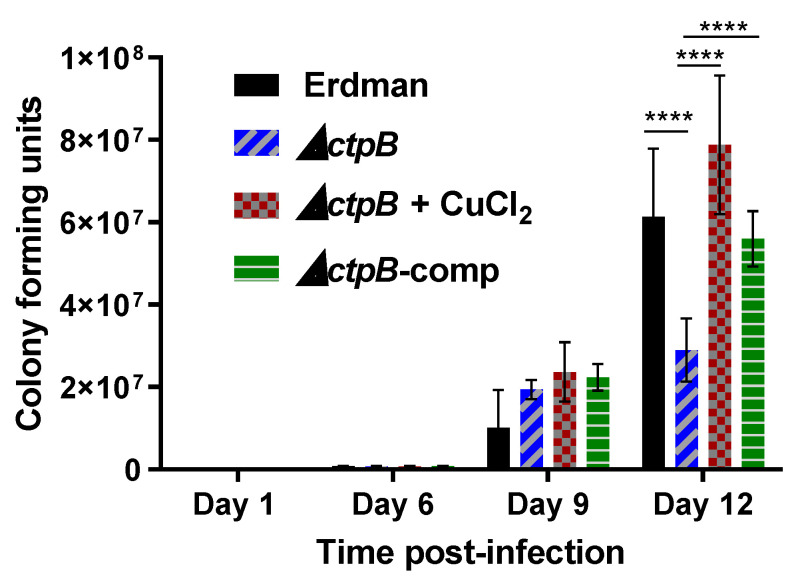
*ctpB* is required for replication and copper acquisition by *M. tuberculosis* in adipocytes. 3T3-L1 adipocytes were infected for 24 h at MOI = 1 while the indicated strains were cultured in DMEM-FBS (basal medium, BM) or BM supplemented with 0.1 mM CuCl_2_. At the indicated time-points, cells were lysed with 1% Triton X-100 and viable bacteria were enumerated by plating on 7H10tgADS agar. Results shown are the averages with standard error from two experiments performed in triplicate. ANOVA with multiple comparisons between groups was used to assess significant differences between groups (**** *p* < 0.0001).

We next examined whether *ctpB* is required for *M. tuberculosis* replication in a copper-restricted host cell. The high hydrophobic content of lipid droplets within adipocytes presents a natural barrier against the free diffusion of copper ions. Both *M. tuberculosis* and *M. canettii* have been shown to replicate within murine primary and 3T3-L1 adipocytes [23], and *M. tuberculosis* has been detected within adipose deposits of infected mice [24]. We observed no differences in the replication of Δ*ctpB* versus the wild-type parent Erdman or the complemented mutant nine days after infection of 3T3-L1 adipocytes. However, by 12 days post-infection, the mutant developed a growth defect resulting in significantly fewer bacteria relative to the control strains (Figure 4). The growth defect of the mutant was abrogated in adipocytes cultured in medium containing 0.1 mM copper chloride (Figure 4). The effects of metal supplementation on the expression of *ctpB* in adipocytes infected with the strain Erdman were also examined. Transcription of the gene was elevated two-fold in basal medium relative to copper-supplemented conditions, while zinc had no effect (Figure S1B). Thus, *ctpB* is needed for copper acquisition by *M. tuberculosis* for optimal growth within adipocytes.

### 2.5. Loss of CtpB Increases M. tuberculosis Virulence in DBA/2 Mice

**Figure 5:**
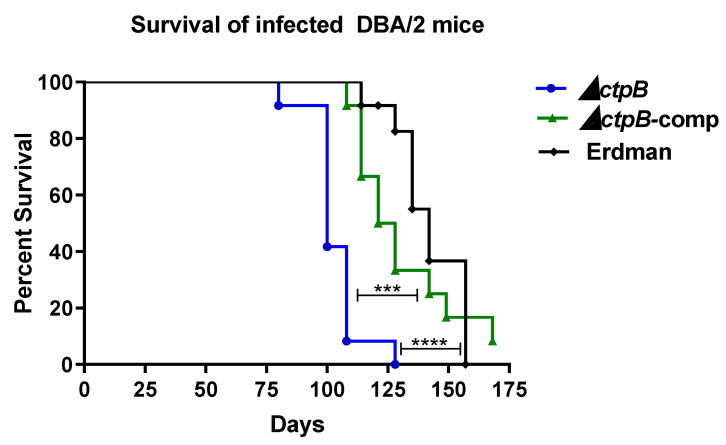
Deletion of *ctpB* from *M. tuberculosis* increases virulence in DBA/2 mice. Shown are Kaplan–Meier curves for relative survival of DBA/2 mice intravenously infected with the *M. tuberculosis ctpB* mutant (Δ*ctpB*), parent strain Erdman, or complemented mutant (Δ*ctpB*-comp). When moribund, animals were euthanized. Log-rank (Mantel–Cox) test was used to assess significant differences between groups (*** *p* < 0.001, **** *p* < 0.0001).

As our data support *ctpB* function in copper acquisition when availability is limited, we next examined if it impacts *M. tuberculosis* virulence. Groups of DBA/2 mice were intravenously infected with 1–2 × 10^5^ CFU of the *ctpB*-deletion mutant, the complemented mutant, or parent strain Erdman. Day-one confirmation of infection indicated that 1450, 1600, and 2430 CFU were present in the lungs of mice infected with strains Δ*ctpB*, Δ*ctpB*-comp, and Erdman, respectively. The remaining mice were monitored for long-term survival. Surprisingly, the median survival time of mice infected with strain Erdman was 42% longer than that of mice infected with the *ctpB* mutant (142 versus 100 days, respectively) (Figure 5). Mice infected with the *ctpB*-complemented mutant had an intermediate median survival time (124.5 days). An examination of the lungs at the time of death revealed extensive pathology at both the gross and microscopic levels in all groups and no significant differences in blinded pathology scores (Figures S3 and S4). Equivalent *M. tuberculosis* burdens between strains were observed in the lungs (10^7^ CFU), spleen (10^5^ CFU), and liver (10^5^ CFU) (Figure S5). The accelerated death of mice infected with the *ctpB* mutant without differences in organ burdens or lung pathology among all groups at the time of death is consistent with the mutant replicating more quickly than the parent Erdman. Thus, the loss of the ability to uptake copper at low concentrations correlates with increased survival and replication in this infection model system.

### 2.6. Conservation of CtpB Only among Intracellular Pathogenic Mycobacteria

**Figure 6:**
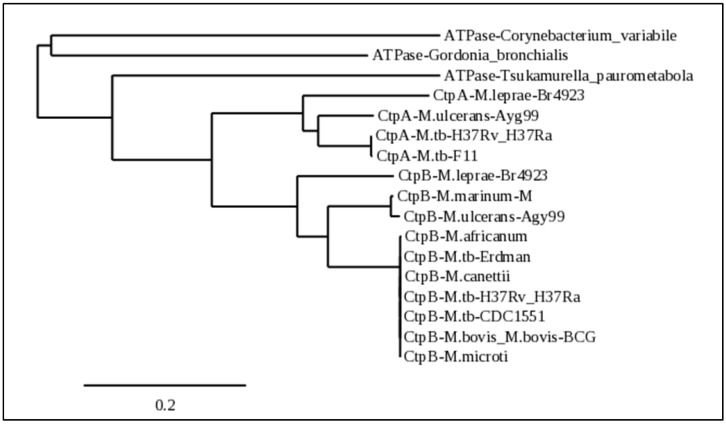
CtpB conservation among intracellular pathogenic *Mycobacterium* species. The phylogenetic tree was rendered with Phylogeny (http://www.phylogeny.fr/ (accessed on 2 April 2022)), the alignment of CtpB homologs from mycobacterial strains of interest and top hits of homologs were obtained through the Mycobrowser precomputed nonredundant protein BLAST search results for the *M. tuberculosis* H37Rv CtpB. Representative homologs for the *M. tuberculosis* (M.tb) complex, *M. marinum*/*M. ulcerans*, and *M. leprae* clades are shown. Branches with a support value less than 50% are collapsed. Genbank entries used: NP_214617.1, WP_003400797.1, YP_004743591, CCC25178.1, WP_012392262.1, WP_011742338.1, CAR72097.1, CAA86363.1, WP_011961619.1, NP_214606.1, MWP_003400679.1, WP_014000034.1, WP_012392258.1, YP_908222.1, CAR72084.1, ACY21849.1, ADG76735.1.

While previous studies have demonstrated CtpB as a copper (I)-transporting P-type ATPase with homology to known copper-exporting P-type ATPases [20], our data indicate CtpB functions to support growth in copper-poor environments. Interestingly, CtpB is extensively conserved (99–100% identity) among species in the *M. tuberculosis* complex (MTC) (Figure 6). Outside the MTC, CtpB is next most conserved among members of a clade that includes the mycolactone (ML)-producing species *M. marinum* and *M. ulcerans* (82% identity, 90% similarity), followed by *M. leprae*, which is the etiological agent of leprosy (78% identity, 87% similarity) (Figure 6). The next closest matches are copper (I)-exporting CtpA homologs from MTC members and the ML clade (68–69% identity, 78–80% similarity) (Figure 6). After CtpB and CtpA homologs in the MTC, the ML clade, and *M. leprae*, sequence conservation sharply drops (<60 identity, <71% similarity) and proteins from nonmycobacteria genera (including *Gordonia*, *Tsukamurella*, and *Corynebacteria*) appear (Figure 6). Alignment of the mycobacterial CtpB homologs shows extensive conservation throughout; however, CtpB and CtpA homologs differ at various regions (Figure S6). Not included in this analysis due to low conservation, the top NCBI CtpB BLAST hits in the *Mycobacterium smegmatis* strain mc2-155 (Genbank taxid:246196) have only 49% identity and 63% similarity. Thus, CtpB is extensively conserved among members of the MTC, highly conserved in the *M. marinum*/*M. ulcerans* clade, and conserved in *M. leprae*, but not in other mycobacteria.

## 3. Discussion

The essentiality of copper for the activity of key redox enzymes and the potential toxicity of free copper ions necessitate networked systems for sensing, regulating, importing, and chaperoning this highly reactive metal to cuproenzymes, storage molecules, or efflux pumps, depending on copper levels. The hydrophobic character of biological membranes prevents the free diffusion of polar molecules. For copper traversal of these barriers in bacteria, pore-forming channels can be formed in the outer membrane, or substrate-specific transport systems can be utilized in either membrane. A family of related outer-membrane porin proteins MspA, MspB, and MspC in *M. smegmatis* were found to function in copper acquisition. The growth defect of a *M. smegmatis mspA mspC* mutant in trace copper conditions was abrogated by introducing *mspA*, *mspB*, or *mspC*; moreover, the heterologous overexpression of *mspA* in *M. tuberculosis* increased copper sensitivity and production of the periplasmic multicopper oxidase, MmcO, which oxidized Cu (I) to the less reactive Cu (II) state [25]. How copper traverses the outer membrane of *M. tuberculosis* remains unclear. This pathogen lacks homologs of the MspA family, while the deletion of *Rv1698*, which encodes an identified outer-membrane channel protein MctB, increased copper sensitivity, supporting its function in copper export rather than import [26].

When abundant, copper is likely imported across the cytoplasmic membrane through ubiquitous major-facilitator superfamily (MSF)-type transporters, which utilize energy from the proton gradient to selectively transport a wide range of substrates [27]. The range of substrates transported by the thirty MSF family homologs identified in *M. tuberculosis* have not been studied in detail; however, most have been linked to antibiotic resistance [28]. Members of the MSF family appear to have low substrate specificity and affinity, allowing the transport of a variety of substrates [29]. The presence of glutathione and other reductants in the bacterial cytosol reduce copper (II) to copper (I) [30].

Cytoplasmic levels of free copper are kept low in part by copper-storage proteins, such as MymT in *M. tuberculosis*, which binds up to six copper (I) ions [31], and copper-efflux pumps. Bacteria have two types of P-type ATPases that couple copper (I) export to ATP hydrolysis: the CopA1 family comprises low-affinity, high-turnover transporters that function to efflux excess copper, while the CopA2 family comprises high-affinity, low-turnover pumps that export copper (I) to the periplasm for insertion into cuproenzymes, and some species have multiple CopA2 proteins that export to different cuproenzymes [32]. Of the three *M. tuberculosis* genes encoding copper-transporting P-type ATPases, the transcription of *ctpV* was upregulated 150-fold in response to 0.5 mM copper, while *ctpA* and *ctpB* were slightly downregulated, and the deletion of *ctpV* increased copper sensitivity [19]. These observations support CtpV as a CopA1-type pump for rapid copper efflux. CtpA is a probable CopA2-type exporter for copper delivery to cuproenzymes. The expression of *M. tuberculosis ctpA* in *M. smegmatis* enhanced copper tolerance and increased ATPase activity of the membrane fraction only in response to copper (I) [33]. When *M. tuberculosis* bacilli encounter a high-copper environment, it alleviates the need for a high-affinity CopA2-type copper (I) exporter for insertion into cuproenzymes, such as the cyt *aa3-bc1* respiration supercomplex.

Our data are consistent with the function of CtpB as a copper-importing P-type ATPase or as a high-affinity CopA2-type exporter to more-efficiently deliver limited cytoplasmic copper to the essential cyt *aa3-bc1* respiration complex. CtpB functioning as a copper importer would be rare as most bacterial copper-transporting P-type ATPases export copper (I) from the cytoplasm [32,34]. However, Lewinson and colleagues reported HmtA as a P-type ATPase in *Pseudomonas aeruginosa* that increases intracellular copper and zinc pools [35] (REF). The gradual development of growth defects in *ctpB* mutants after serial culture in copper-starved medium and reversal of the defects by copper supplementation (Figure 3), and the reduced replication of the *M. tuberculosis ctpB* mutant in adipocytes in the absence of copper supplementation (Figure 4) support either hypothesis.

**Figure 7:**
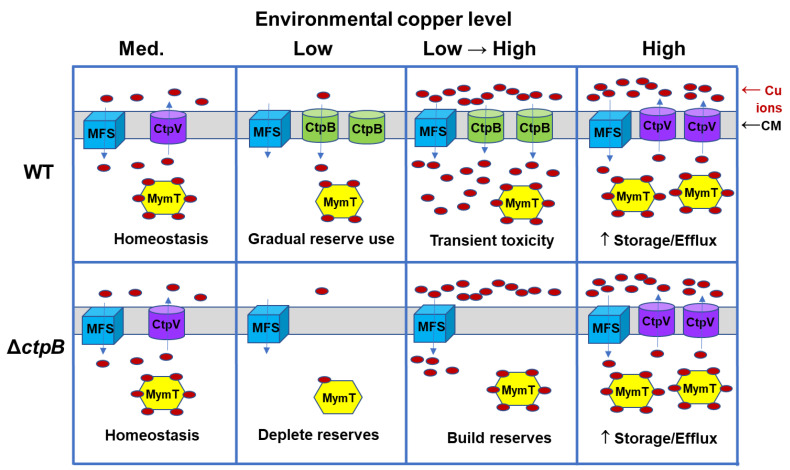
Model for CtpB function in *M. tuberculosis* copper uptake in various copper environments. Bacteria use major-facilitator superfamily (MSF) transporters to selectively import a wide range of divalent metals, including copper, across the cytoplasmic membrane (CM) using proton-motive force. Which *M. tuberculosis* MSF proteins function in copper uptake is unknown, but this pathogen is known to produce cytoplasmic metallothionein (MymT) to store intracellular copper ions and copper-efflux P-type ATPase CtpV for homeostatic control of intracellular copper levels. In a copper-poor environment, the copper (I)-import P-type ATPase CtpB is made to increase copper-uptake capacity, while *ctpV* expression is inhibited to minimize efflux. If the external environment rapidly changes from low to high copper, such as when a mycobacteria-containing phagosome fuses with a lysosome, the high capacity to import copper may be more detrimental to wild-type (WT) *M. tuberculosis* bacteria if copper import exceeds capacity for storage and efflux. Bacteria that survive respond to the elevated levels by increasing production of CtpV and MymT to restore copper homeostasis. In contrast to the WT*,* a Δ*ctpB* mutant is predicted to more quickly draw down its copper reserves in a low-copper niche, but restore its reserves without importing excess copper in a copper-toxic environment. Increased resistance to copper toxicity by Δ*ctpB* may enable the mutant to replicate to higher levels than the WT in mice, resulting in accelerated manifestation of disease pathology and host death.

How does the loss of the ability to import or direct copper by the *ctpB* mutant agree with the seemingly paradoxical increase in virulence and replication in the murine model? If CtpB enables *M. tuberculosis* to import copper, it may contribute to copper overload within phagosomes. Cytosolic copper homeostasis relies on the balancing of copper import, storage, and export. Within a host, the pathogen will encounter environments with varied copper content, as illustrated with a simplistic model in Figure 7. In a medium-copper niche, CtpB function is not required, as ample copper enters through MSF transporters, with some of the metal stored by binding to MymT, and the excess exported via CtpV (Figure 7, column 1). In a low-copper environment, low-affinity MSF transporters are ineffective and production of CtpV is downregulated, while production of CtpB would occur to enable the import of scarce copper ions. Copper stores may begin to be used, but at a faster rate by a *ctpB* mutant (Figure 7, column 2). A rapid transition from low to high copper may occur upon phagocytosis by an activated macrophage. Neither the wild-type nor the mutant strain would have time to synthesize new CtpV, but only the wild-type strain expresses CtpB, resulting in copper intoxication due to influx through both MSF and CtpB, with subtoxic influx occurring in the *ctpB* mutant (Figure 7, column 3). To adapt to the high-copper environment, CtpB production would cease and CtpV and MymT production would increase for export and storage of the metal, respectively (Figure 7, column 4). Bacteria that escape the phagosome have copper reserves to sustain the pathogen, as it again encounters copper-restricted niches. If CtpB instead functions to export copper to the cyt *bc1-aa3* supercomplex, then increased respiration would be expected upon the rapid shift to a high-copper phagosomal environment, which could result in enhanced production of reactive oxygen species.

Our findings are in support of CtpB function in copper import or directing copper to the *cyt bc1-aa3* complex and are consistent with the published *ctpB* data. While León-Torres and colleagues examined the effects of *M. tuberculosis ctpB* expression in *M. smegmatis* and observed enhanced copper resistance [20], we observed copper sensitivity (Figure 1). How can this be rationalized? A critical difference in our approach was the placement of the gene under control of an aTC-inducible promoter rather than the constitutive Hsp60 promoter. From a copper-scavenging perspective, the prolonged constitutive expression of *ctpB* resulting in increased cytosolic copper would be sensed by the bacteria and lead to the upregulation of copper-efflux and detoxification mechanisms concomitant with the reported copper tolerance [20]. In contrast, inducing *ctpB* with aTC at the same time as copper supplementation enabled growth phenotypes to manifest before the bacteria had time to adapt to the elevated copper influx (Figure 1). The lack of growth effects associated with the *M. tuberculosis* H37Ra *ctpB* mutant at varied copper levels [20] is expected if the bacteria have not depleted internal copper stores. Growth defects of our *ctpB* mutants of *M. tuberculosis* Erdman and *M. bovis* BCG only manifested after serial subculture in copper-deficient medium (Figure 3). If we consider the phenotypes from the perspective of CtpB functioning to efficiently export scarce copper to respiration complexes, then increased reactive oxygen radicals would likely be generated when *ctpB* is overexpressed, resulting in increased toxicity. Thus, the observations from both studies are consistent with CtpB functioning in either copper import or for directed export to cyt *bc1-aa3* supercomplex.

Phylogenetically, CtpB is highly conserved only among intracellular pathogenic *Mycobacterium* species (MTC, mycolactone clade, and *M. leprae*). It is also noteworthy that these same groups also have a closely-related copper (I)-exporting CtpA homolog. The evolution of a high-affinity copper (I)-importing function from the duplication and mutation of *ctpA* correlates with the enhanced ability of these mycobacteria to adapt to host nutritional immunity. In a copper-starved environment, these pathogens will eventually develop cytosolic reductive stress, which may lead to a greater reducing environment in the periplasm, where copper (II) may be reduced to copper (I) for import by CtpB, so that the metal can be more efficiently delivered to CtpA for export to the cyt *aa3-bc1* respiration supercomplex. Alternatively, CtpB evolved as a second high-affinity CopA2-type exporter that outcompetes CtpA for cytoplasmic copper for directed export to the essential cyt *aa3-bc1* respiration supercomplex, which would suggest that CtpA functions to transfer the metal to the supercomplex and to other cuproenzymes.

Structural and mutational analyses will help elucidate CtpB function and to identify chaperones that participate in copper trafficking in intracellular pathogenic mycobacteria. If CtpB imports copper, it will be particularly interesting to understand how the Post–Albers model for P-type ATPase function is influenced by the binding of a periplasmic copper chaperone. Additionally, in light of the essentiality of copper for *M. tuberculosis* aerobic respiration, examination of the *ctpB* mutant in an animal model that develops hypoxic granulomas will be informative. This human physiological niche is absent in most murine models, but is generated in response to *M. tuberculosis* infection of nonhuman primates, rabbits, guinea pigs, and C3HeB/FeJ (Kramnik) mice [36,37].

## 4. Materials and Methods

### 4.1. Bacterial Strains and Growth Conditions

Strains of mycobacteria were initially cultured in Middlebrook 7H9 broth or 7H10 agar (Thermo Scientific) supplemented with 0.5% glycerol, 0.05% Tween 80, and albumin–dextrose–saline (ADS) or oleic-acid–albumin–dextrose–saline (OADS) enrichment [38]. For copper-response studies, strains were cultured in copper-free Sauton medium supplemented with 0.05% tyloxapol (SMT) [39] or SMT supplemented with the indicated concentration of the copper (II) chelator tetrathiomolybdate (TTM) and/or copper salts at concentrations that were consistent with those reported for inactivated (limiting) or activated (inhibitory) intraphagosomal environments [11]. Plasmids, strains, and primers used in these studies can be found in Tables S1–S3, respectively. Transformation of *Mycobacterium* species by electroporation employed standard techniques [37]. Cultivation of *E. coli* strains used Luria–Bertani broth and agar. When required, kanamycin was used at 50 µg/mL for *E. coli* and 25 µg/mL for mycobacteria. Hygromycin was used at 50 µg/mL and gentamycin at 20 µg/mL in either *E. coli* or mycobacteria. For induced expression of genes controlled from anhydrotetracycline-controlled promoters, the drug was used at the subclinical concentrations indicated.

Controlled expression of *M. tuberculosis* genes in mycobacterial recipient host strains utilized the plasmid pOSN20. It was derived from plasmid pSR173 that was used previously for inducible biosynthesis of *M. tuberculosis* SigC with an N-terminal *myc* tag under control of the anhydrotetracycline-inducible promoter, P*_myctetO_* [22]. Plasmid pOSN20 was constructed by annealing primers P1705 and P1706 to yield the *E. coli lacZ* promoter lacking the binding site for the catabolite-activator protein but containing the *lac* operator site. The annealed fragment was ligated into the *Sph*I site of pSR173 upstream of the mycobacterial P_tb21_ promoter preceding the tetracycline repressor gene, *tetR(B)*. This was performed to limit leaky expression in *E. coli* of genes inserted into pOSN20 to replace *sigC*. Plasmid pOSN19 was generated by removal of *sigC* from plasmid pOSN20 by digestion with *Sph*I and *Nsi*I, blunting the DNA ends and re-ligating the vector. Plasmid pOSN37 was produced by PCR of *M. tuberculosis ctpB* with primers P2059 and P2041 and insertion into pOSN20 digested with *Pac*I and *Nsi*I to replace *sigC*, yielding a plasmid encoding a Myc-tagged CtpB translational fusion protein. Plasmid pOSN38 was similarly constructed by amplification of *M. tuberculosis ctpV* with primers P2060 and P2061 and insertion into the pOSN20 digested with *Pac*I and *Nsi*I. DNA sequencing confirmed the absence of mutations in the inserted genes. Plasmid pOSN26 was produced by PCR of *M. tuberculosis ctpV* with primers P1880 and P1881 and insertion pOSN20 after removal of *myc-sigC* by *Sca*I/*Nsi*I digestion. Plasmid pOSN30 was similarly made by PCR of *ctpB* with primers P1605 and P1606 and insertion into the *Sca*I/*Nsi*I-digested pOSN20.

For deletion of *ctpB* from *M. tuberculosis* and from *M. bovis* BCG, plasmid pOSN21 was created by sequential insertion of PCR products from ~1-kb genomic regions upstream and downstream of *ctpB* in *M. tuberculosis,* generated with primer pairs P2042/P2043 and P2044/P2045, respectively, into the *Afl*I-*Xba*I and *Nco*I-*Bgl*II regions of pYUB854 flanking the *hyg* cassette. The Δ*ctpB::hyg* allelic exchange substrate (AES) for deleting *ctpB* through recombineering was obtained by digestion of pOSN21 with *Bgl*II and *Xba*I. For *ctpB* complementation, plasmid pOSN5 was generated by PCR of the *M. tuberculosis ctpB* region from 209 bp upstream to 49 bp downstream with primers P1575 and P1576 and insertion into the *Hin*dIII site of plasmid pMV306. Plasmid pOSN40 was produced by replacement of the kanamycin-resistance gene of pOSN5 with the gentamycin-resistance element obtained from plasmid pPR27 by PCR with primers P1250 and P2251 and insertion into the *Nhe*I-*Spe*I region of pOSN5.

Deletion of *ctpB* from *M. bovis* BCG and *M. tuberculosis* utilized mycobacterial recombineering [39]. In brief, parent strains were transformed with plasmid pJV53 encoding mycobacteriophage Che9 recombinase proteins gp60 and gp61 under control of an acetamide-inducible promoter [40]. The Δ*ctpB::hyg* AES was electroporated into competent cells prepared after 12–16 h of growth in the presence of acetamide; hygromycin-resistant transformants were screened by PCR.

Strains of *M. tuberculosis* used for infection experiments were passaged three times in SMT and stored in 1 mL aliquots at −80 °C upon reaching OD_600_ = 1. To determine the bacterial counts of the *M. tuberculosis* infection stocks, all strains were thawed at 37 °C, harvested by centrifugation (10,000× *g*, 5 min), resuspended in phosphate-buffered saline (PBS), and sonicated in an ultrasonic water bath twice for 30 s. Serial dilutions of each strain were performed in PBS + 0.05% Tween 80 and plated onto 7H10ADStg agar. Colonies were enumerated after 3 to 4 weeks of incubation at 37 °C to determine the titers of the infection stocks.

### 4.2. Culture and Infection of Cells Lines

3T3-L1 fibroblast cells (ATCC^®^ CL-173) were cultured in DMEM basal medium supplemented with 10% heat-inactivated bovine calf serum (DMEM-BCS) at 37 °C and 5% CO_2_ and replaced every three days with fresh pre-warmed medium. Differentiation of pre-adipocytes to adipocytes was started two days after confluency by stimulation of cells with 0.5 mM 3-isobutyl-1-methylxanthine (IBMX), 0.25 μM dexamethasone, 2 μM rosiglitazone, and 1 μg/mL insulin in DMEM plus 10% heat-inactivated fetal bovine serum (DMEM-FBS) using the methodology of Zebisch and colleagues [41]. Two days later, the medium was replaced with DMEM-FBS with 1 μg/mL insulin. Adipocytes were maintained in DMEM-FBS that was changed twice per week. Adipocytes were infected at MOI = 1 using infection stocks prepared for the indicated *M. tuberculosis* strains and cultured in DMEM-FBS alone or medium supplemented with 0.1 mM CuCl_2_ or ZnCl_2_. After 24 h incubation at 37 °C, monolayers were washed three times with PBS and fresh medium was applied. At the indicated time points, cells were lysed with 1% Triton X-100. To enumerate viable bacteria, cell lysates were serially diluted in phosphate-buffered saline (PBS) + 0.05% Tween 80, plated onto 7H10ADStg, and colonies counted after a 3-to-4-week incubation at 37 °C.

### 4.3. RNA Isolation and Quantitative RT-PCR

For RNA isolation from in vitro bacterial cultures, *M. tuberculosis* strain Erdman was passaged three times in SMT, then replicate cultures were grown in SMT inoculated to OD_600_ = 0.05. At OD_600_ = 0.35, TTM was added to 20 µM to the half of the cultures. At OD_600_ = 0.45, bacteria were harvested by centrifugation (10,000× *g*, 5 min) and the pellets stored at −80 °C. RNA was isolated as described previously [16]. For RNA isolation from the *M. tuberculosis*-infected 3T3-L1 adipocytes, host cells infected at MOI = 1 were cultured in basal medium (DMEM-FBS 10%) alone or with addition of either 0.1 mM CuCl_2_ or ZnSO_4_ and incubated at 37 °C, 5% CO_2_ with medium replaced every 3 days. At day 10 post-infection, cells were lysed by suspension in 4 M guanidine isothiocyanate and bacteria harvested by centrifugation (10,000× *g*, 45 min). Bacterial RNA was isolated from the pellets using the methods described earlier for isolation from axenic cultures. For cDNA synthesis, 0.5 µg RNA was used with 200 ng random hexamers and Superscript III Reverse Transcriptase (Invitrogen) according to the manufacturer instructions. Quantitative PCR was performed using SYBR Green PCR Master Mix (Applied Biosystems), gene-specific primers, and prepared cDNA on a BioRad iCyler. For ΔΔCt analysis, gene expression was normalized to the *M. tuberculosis* housekeeping gene *sigA* [16].

### 4.4. Murine Infection Studies

Groups of female, 8-week-old, DBA/2J mice (Jackson Laboratories, Bar Harbor, ME 04609) mice were intravenously infected via tail vein injection with 1–2 × 10^5^ CFU *M. tuberculosis* strain Δ*ctpB*, the complemented mutant (Δ*ctpB*-comp), or Erdman. One day after infection, the mycobacterial lung burden was enumerated for one mouse in each group as follows: the lungs were harvested post-euthanasia, homogenized and plated on Middlebrook 7H11 agar supplemented with 10% ADS, 0.5% glycerol, and 0.05% Tween 80 (7H11gtADS), and resulting colonies counted after a 3-to-4-week incubation at 37 °C. The health of all animals was monitored daily, and mice were weighed weekly. Mice were euthanized when moribund. At necropsy, gross pathology of the lungs was examined. Half of the lungs were fixed in 10% neutral-buffered formalin for histopathological assessment, while the other half of the lungs as well as the spleen and liver were homogenized in PBS–Tween and serial dilutions plated on 7H11tgADS agar and bacterial counts enumerated after a 3-to-4-week incubation at 37 °C. For histopathology, formalin-fixed lungs were routinely processed for paraffin embedding, sectioning at 4 µm thickness, and staining with hematoxylin and eosin. Lung slides were assessed by a board-certified, veterinary pathologist using the following scoring system. Neutrophil (PMN) score: 1 = up to 25% of alveolar infiltrate composed of neutrophils, 2 = 26–50%, 3 = 51–75%, 4 = more than 75%; necrosis, alveolar, and alveolar edema scores: 1 = focal, 2 = multifocal, 3 = coalescing, 4 = diffuse; Vasculitis score: 1 = leukocytes infiltrating vessel wall, 2 = changes in 1 + clear spaces separating smooth muscle cells, 3 = changes in 2 + fibrinoid change, 4 = vessel wall effacement; perivascular cuffing (PVC) score: 1 = 1 layer of leukocytes surrounding vessel, 2 = 2–5 cells thick, 3 = 6–9 cells thick, 4 = 10 or more cells thick.

### 4.5. Bioinformatics

Sequences with homology to CtpB from *M. tuberculosis* strain H37Rv were identified by a NCBI protein BLAST search launched through the Mycobrowser server (https://mycobrowser.epfl.ch (accessed on 2 April 2022)). The searched database consisted of all non-redundant GenBank CDS translations + PDB + SwissProt + PIR + PRF excluding environmental samples from WGS projects. Additional CtpB homologs from mycobacterial strains of interest were obtained from Genbank. Sequences of representative homologs from various species were aligned using Phylogeny (http://www.phylogeny.fr/index.cgi, accessed on 2 April 2022) to generate a phylogenetic tree. Colorized alignments of the mycobacterial strains used the phylogenetic tree were obtained by first aligning with Phylogeny and then pasting the fasta format alignment output into the Sequence Manipulator Site (https://www.bioinformatics.org/sms2/index.html, accessed on 2 April 2022).

### 4.6. Statistical Analyses

Statistically significant differences between groups in bacterial growth studies utilized ANOVA with pair-wise comparisons. For the animal-survival comparisons, a log-rank (Mantel–Cox) test was applied to the Kaplan–Meier curves. All statistical analyses were performed using GraphPad Prism software. Differences between groups were deemed significant when *p* < 0.05.

## 5. Conclusions

Our data indicate that CtpB, as a copper transporter, is required only in environments where copper is scarce. The conservation of CtpB exclusively among obligate intracellular mycobacteria is consistent with the evolution of pathogenic species that are able to counter copper nutritional-immunity mechanisms, thereby supporting respiration and other copper-dependent enzyme functions within a host. The elucidation of novel copper-acquisition systems by these important pathogens may reveal Achilles’ heels that can be targeted to develop new antibiotics to aid in the battle to eradicate tuberculosis.

## Data Availability

Data supporting reported results can be found in the Supplementary Materials.

## Supplementary Materials

The following supporting information can be downloaded at: https://www.mdpi.com/article/10.3390/ijms23105713/s1.
