# Supplementary material for: CtpB Facilitates Mycobacterium tuberculosis Growth in Copper-Limited Niches
Source: Int J Mol Sci. 2022 May 20;23(10):5713. doi: 10.3390/ijms23105713 (PMC9147137; doi:10.3390/ijms23105713)
Supplement: Supplementary file 1 [file ijms-23-05713-s001.zip › ijms-1707244-supplementary.pdf]

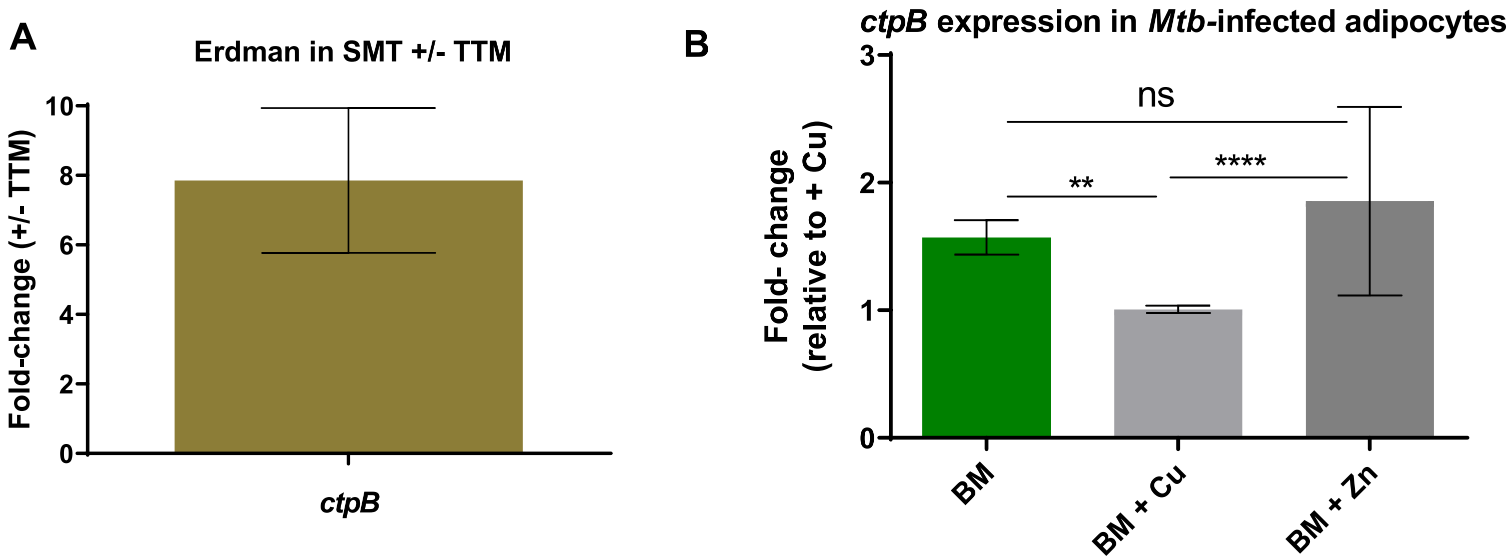

Figure S1. Transcription of *ctpB* in *M. tuberculosis* Erdman increases in response to copper limitation. Gene expression of *ctpB* was examined by quantitative RT-PCR after culture of strain Erdman in A) STM +/- 20 μM TTM or B) after infection of 3T3-L1 adipocytes cultured DMEM-FBS (basal medium, BM) or BM supplemented with 0.1 mM $CuCl_2$ or $ZnCl_2$. Gene expression was normalized to *sigA*.

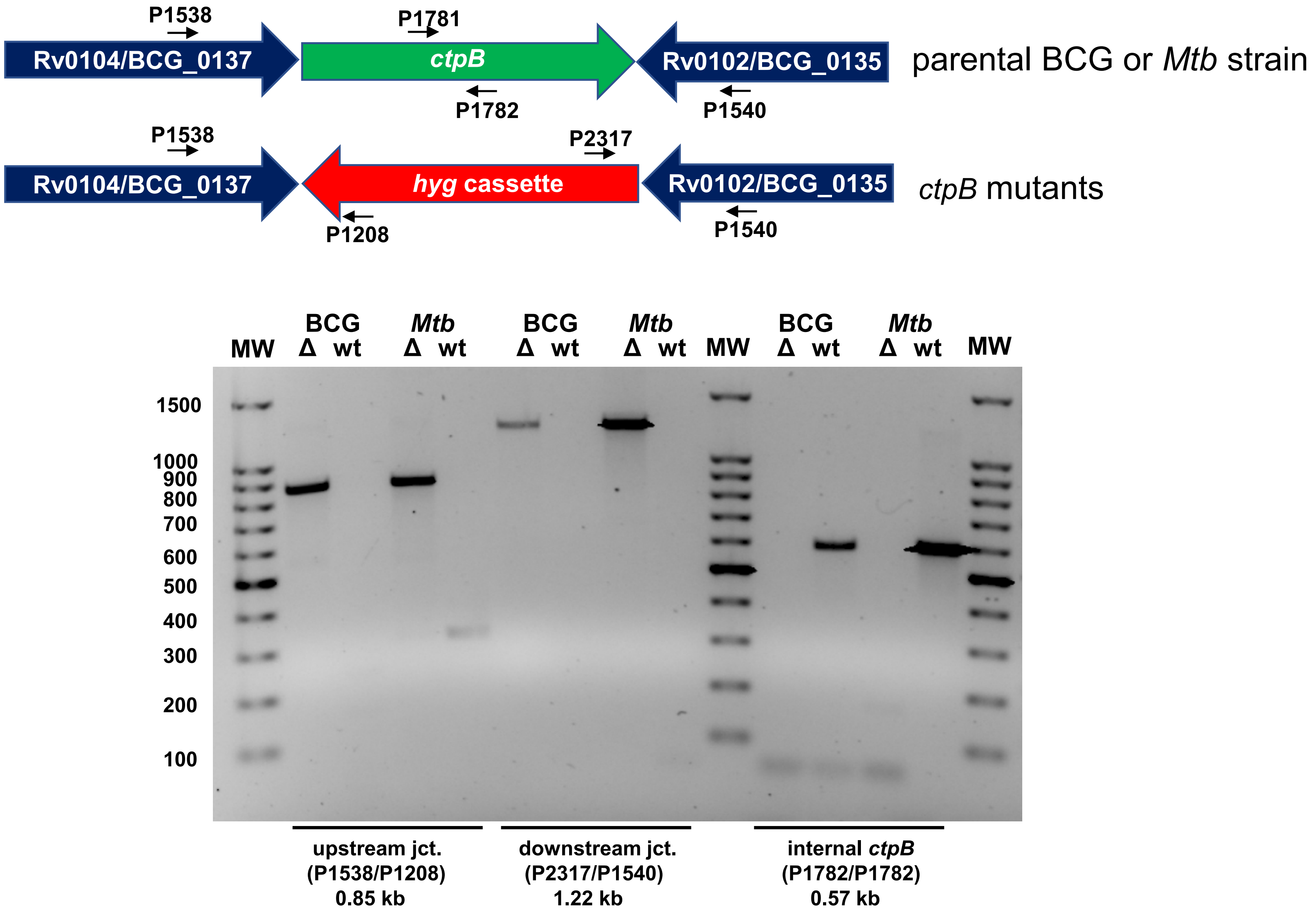

Figure S2. Confirmation of *ctpB* deletion mutants. (Top) Drawing of conserved (99% identical) *ctpB* genomic *M. bovis* BCG and *M. tuberculosis* (*Mtb*) before and after replacement of *ctpB* with a hygromycin-resistance (*hyg*) cassette. Primers used for PCR are indicated (small arrows). (Bottom) Resolution of PCR products on a 1.4% agarose gel. Expected band sizes for the inserted *hyg* cassette in a *ctpB* mutant (Δ) is indicated, as is the expected size of an internal *ctpB* product in the parent strains. Size standards are Promega 100-bp ladder (MW).

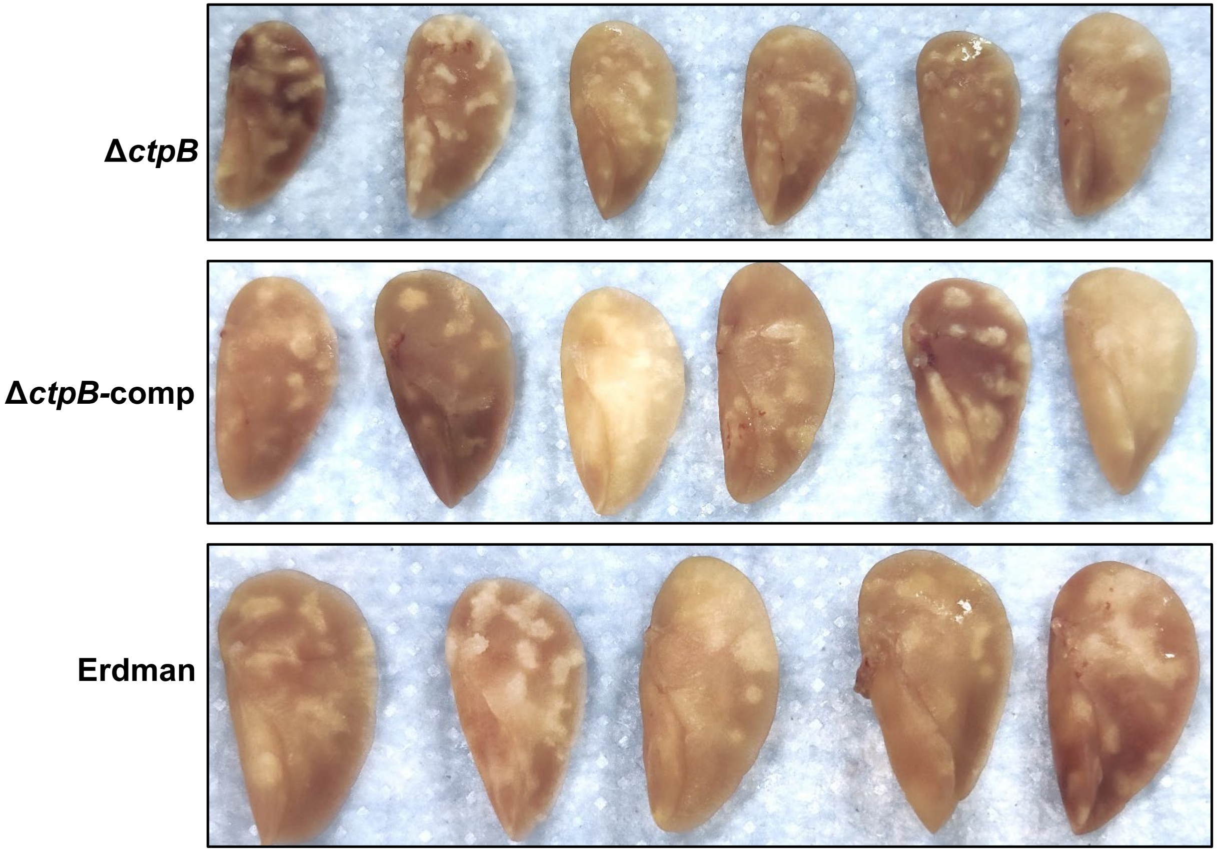

Figure S3. Gross pathology of lungs at time of death is similar for DBA/2 mice infected with Δ*ctpB*, Δ*ctpB*-comp, or *M. tuberculosis* Erdman. After euthanization of infected DBA/2 mice when moribund (Figure 5), the lungs, liver, and spleen were removed. Gross pathology of one lung lobe from each animal is shown. Granulomas are evident by the white foci.

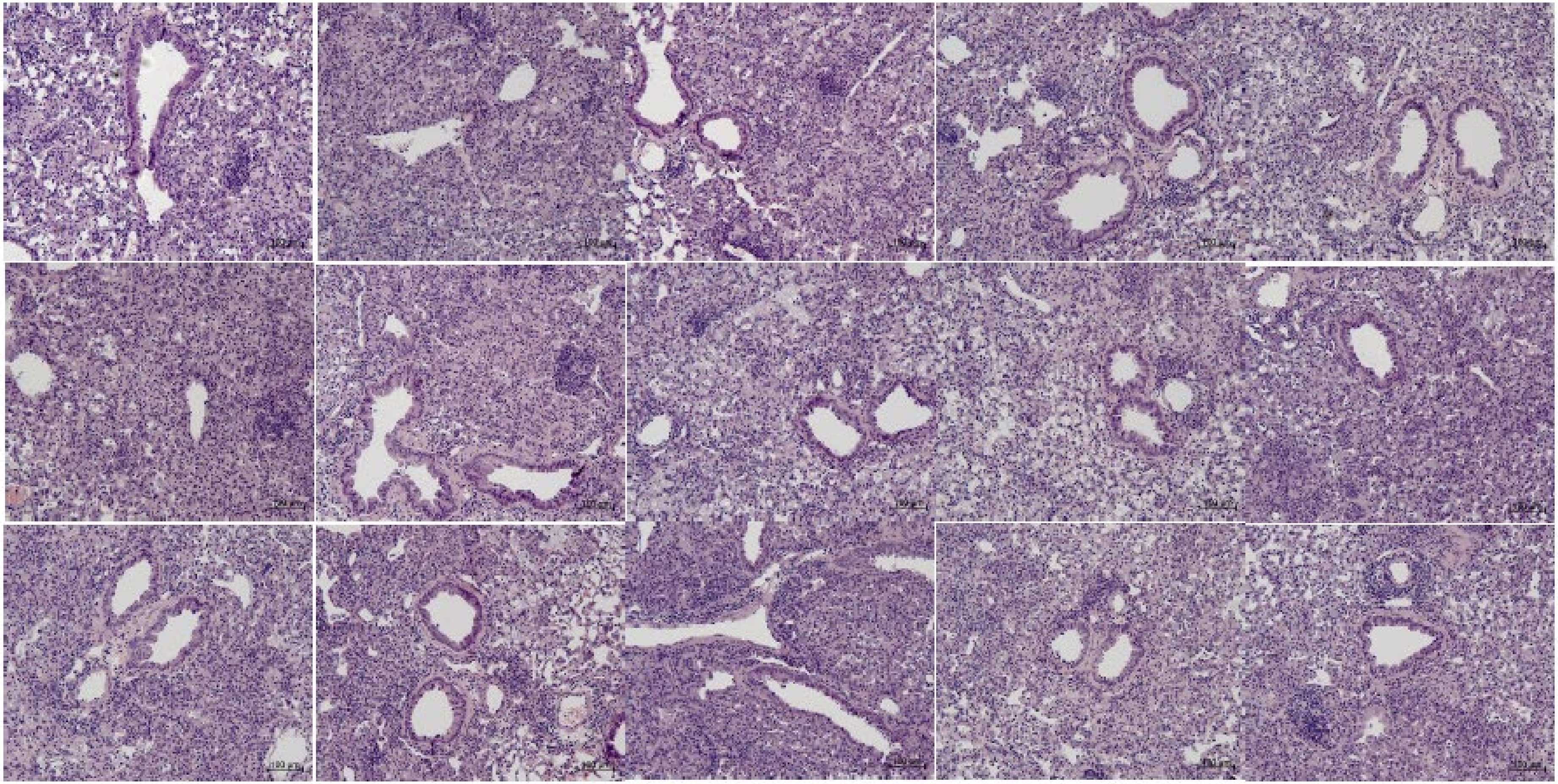

Figure S4. No difference in histopathology of lungs harvested from moribund DBA/2 mice following infection with Δ*ctpB*, Δ*ctpB*-comp, or *M. tuberculosis* Erdman. A) Microscopic visualization of hematoxylin & eosin-stained lung sections from DBA/2 survival study mice infected with strain Δ*ctpB* (top row), Δ*ctpB*-comp (middle row), or *M. tuberculosis* Erdman (bottom row) harvested when animals were moribund.

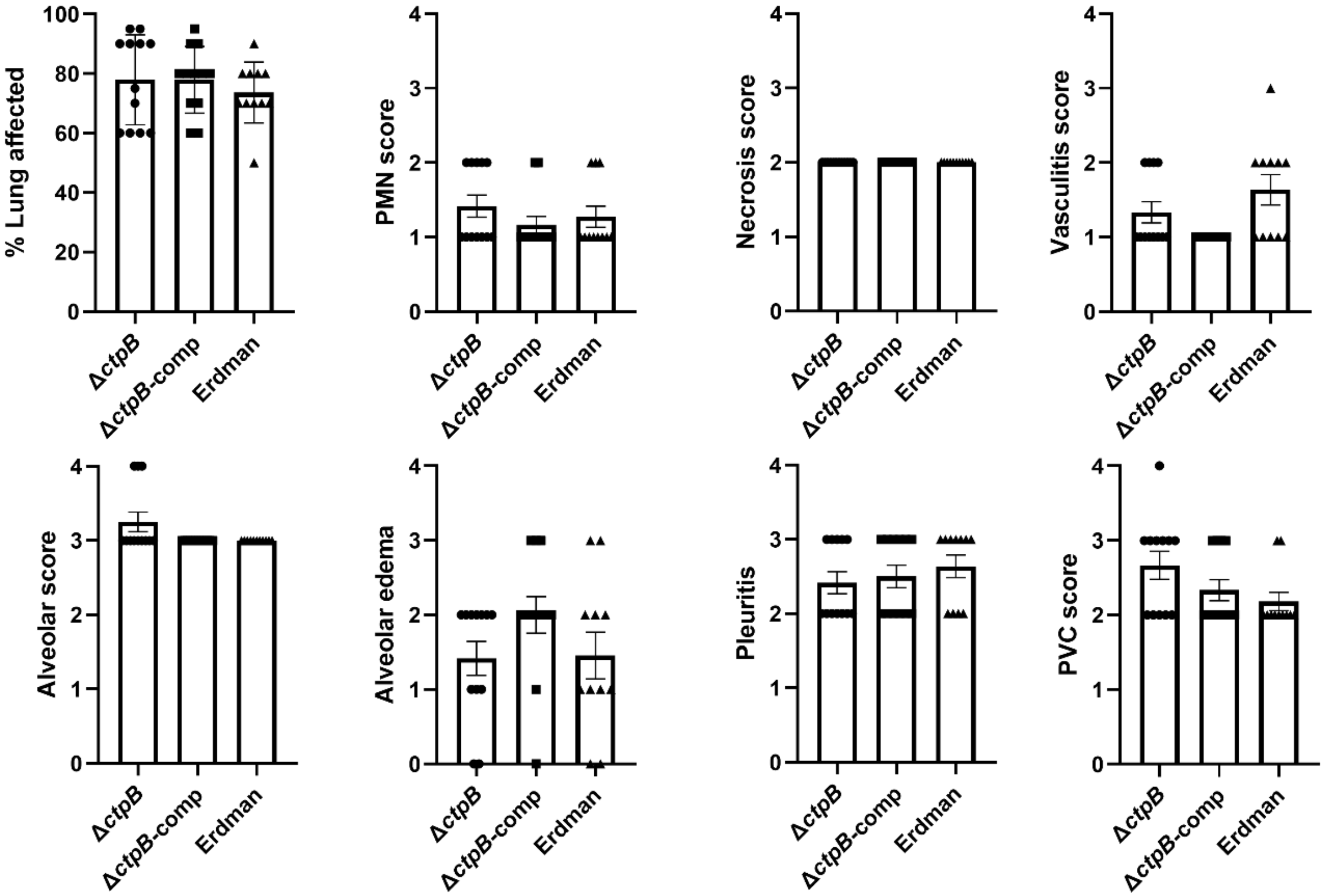

Figure S4 (continued) B) Semi-quantitative histopathological assessments of lung tissues were examined as detailed in the Methods section by a board-certified pathologist. No significant differences in pathology measures at the time of death were observed regardless of the infecting strains.

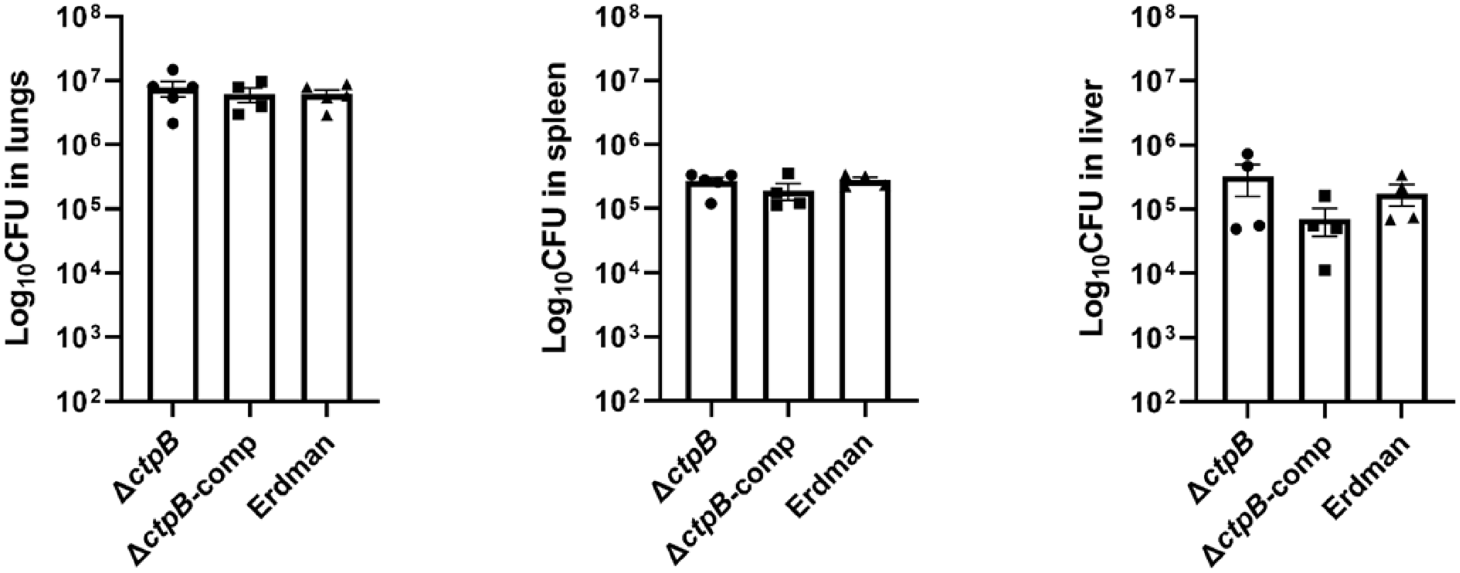

Figure S5. No significant differences in *M. tuberculosis* colony-forming units in the lungs, liver, or spleen at time of death of DBA/2 mice infected with Δ*ctpB*, Δ*ctpB*-comp, or *M. tuberculosis* Erdman. Mycobacterial burdens in DBA/2 mice infected with Δ*ctpB* (circles), Δ*ctpB*-comp (squares), or *M. tuberculosis* Erdman (triangles) were quantified by homogenization and plating lung, liver, and spleen tissues harvested at necropsy of the animal euthanized upon becoming moribund. After counting colonies, no significant differences in mycobacterial counts were observed between infecting strains in the lungs, liver, or spleen.

Figure S6. Alignment of mycobacterial CtpB homologs. Mycobacterial CtpB alignment output generated in Phylogeny was pasted into the Sequence Manipulation Suite (https://www.bioinformatics.org/sms2/color_align_prop.html) set to highlight similar residues present in 70% of the homologs with the same color. The same mycobacterial CtpB homologs used in the phylogram sown on Figure 7 were used. Species/strain names in order of appearance: *M. leprae*, *M. canettii*, *M. tuberculosis* H37Rv, *M. tuberculosis* CDC1551, *M. bovis*, *M. microti*, *M. tuberculosis* Erdman, *M. africanum*, *M. ulcerans*, *M. tuberculosis* F11, *M. tuberculosis* H37Rv.

Color Align Code:

```
G, A, V, L, I
F, Y, W
C, M
S, T
K, R, H
D, E
N, Q
P
```

```
CtpB-Mlepra  -MTASLVEDTNNNH-ESVRRIQLDVAGMLCAACASRVETKLNKIPGVRASVNFATRVATIDAVD-VAVDELRQVIEQAGY 77
CtpB-Mcanet  -MAAPVVGDADL---QSVRRIRLDVSGMSCAACASRVETKLNKIPGVRASVNFATRVATIDAVG-MAADELCGVVEKAGY 75
CtpB-H37Rv   -MAAPVVGDADL---QSVRRIRLDVLGMSCAACASRVETKLNKIPGVRASVNFATRVATIDAVG-MAADELCGVVEKAGY 75
CtpB-CDC1551 -MAAPVVGDADL---QSVRRIRLDVSGMSCAACASRVETKLNKIPGVRASVNFATRVATIDAVG-MAADELCGVVEKAGY 75
CtpB-Mbovis  -MAAPVVGDADL---QSVRRIRLDVSGMSCAACASRVETKLNKIPGVRASVNFATRVATIDAVG-MAADELCGVVEKAGY 75
CtpB-Mmicro  -MAAPVVGDADL---QSVRRIRLDVSGMSCAACASRVETKLNKIPGVRASVNFATRVATIDAVG-MAADELCGVVEKAGY 75
CtpB-Erdman  -MAAPVVGDADL---QSVRRIRLDVSGMSCAACASRVETKLNKIPGVRASVNFATRVATIDAVG-MAADELCGVVEKAGY 75
CtpB-Mafrica -MAAPVVGDADL---QSVRRIRLDVSGMSCAACASRVETKLNKIPGVRASVNFATRVATIDAVG-MAADELCGVVEKAGY 75
CtpB-Mmarin  -MAAPITGDLDL---GSARRIQLDVSGMSCAACASRVETKLNKVPGVRASVNFATRVATIDAVD-VAADQLCELVEKAGY 75
CtpB-Mulcer  -MAAPITGDLDL---GSARRIQLDVSGMSCAACASRVETKLNKVPGVRASVNFATRVATIDAVD-VAADELCELVEKAGY 75
CtpA-Mlepra  -MRAP-NGWNNL----------------------------PNKLSDFSTLVNSATRVAAINLSEVTQTAEVCEAVRRAA- 49
CtpA-Mulcer  MSTTIVTTDKHLERDQQVQRFQLGITGMSCFACARKVQSTLNKVPGVRASVNFGTRTAIVEAGEDVDAAALCEVIQQAGY 80
CtpA-MtbF11  -MTTAVTGEHH----ASVQRIQLRISGMSCSACAHRVESTLNKLPGVRAAVNFGTRVATIDTSEAVDAAALCQAVRRAGY 75
CtpA-H37Rv   -MTTAVTGEHH----ASVQRIQLRISGMSCSACAHRVESTLNKLPGVRAAVNFGTRVATIDTSEAVDAAALCQAVRRAGY 75

CtpB-Mlepra  RATAHAESAVEEI---DPDADYARNLLRRLIVAALLFVPLADLSTMFAIVPTNRFPGWGYLLTALAAPIVTWAAWPFHRV 154
CtpB-Mcanet  HAAPHTETTVLDKRTKDPDGAHARRLLRRLLVAAVLFVPLADLSTLFAIVPSARVPGWGYILTALAAPVVTWAAWPFHSV 155
CtpB-H37Rv   HAAPHTETTVLDKRTKDPDGAHARRLLRRLLVAAVLFVPLADLSTLFAIVPSARVPGWGYILTALAAPVVTWAAWPFHSV 155
CtpB-CDC1551 HAAPHTETTVLDKRTKDPDGAHARRLLRRLLVAAVLFVPLADLSTLFAIVPSARVPGWGYILTALAAPVVTWAAWPFHSV 155
CtpB-Mbovis  HAAPHTETTVLDKRTKDPDGAHARRLLRRLLVAAVLFVPLADLSTLFAIVPSARVPGWGYILTALAAPVVTWAAWPFHSV 155
CtpB-Mmicro  HAAPHTETTVLDKRTKDPDGAHARRLLRRLLVAAVLFVPLADLSTLFAIVPSARVPGWGYILTALAAPVVTWAAWPFHSV 155
CtpB-Erdman  HAAPHTETTVLDKRTKDPDGAHARRLLRRLLVAAVLFVPLADLSTLFAIVPSARVPGWGYILTALAAPVVTWAAWPFHSV 155
CtpB-Mafrica HAAPHTETTVLDKRTKDPDGAHARRLLRRLLVAAVLFVPLADLSTLFAIVPSARVPGWGYILTALAAPVVTWAAWPFHSV 155
CtpB-Mmarin  QAVPHAESAADDR---DPDAEHARTLLRRLLVAAVLFVPLADLSTMFALVPSTRFPGWGYLLTALAAPIVTWAAWPFHSV 152
CtpB-Mulcer  QAVPHAESAADDR---DPDAEHARTLLRRLLVAAVLFVPLADLSTMFALVPSTRFPGWGYLLTALAAPIVIWAAWPFHSV 152
CtpA-Mlepra  ---LCTDGGEALQR-RQADADNARYLLIRLAVAAALFVPLAHLSVMFAVLPSTHFPGWEWMLTALAIPVVTWAAWPFHRV 125
CtpA-Mulcer  QAEVQPDDGQGAG---DSDAEHARHLLIRLAVAAVLFVPLADLSVMFAVVPSTRFTGWEWVLTALALPVVTWAAWPFHRI 157
CtpA-MtbF11  QADLCTDDGRSAS---DPDADHARQLLIRLAIAAVLFVPVADLSVMFGVVPATRFTGWQWVLSALALPVVTWAAWPFHRV 152
CtpA-H37Rv   QADLCTDDGRSAS---DPDADHARQLLIRLAIAAVLFVPVADLSVMFGVVPATRFTGWQWVLSALALPVVTWAAWPFHRV 152
```

```
CtpB-Mlepra    ALRNARYRAASMETLISAGILAATGWSLSTIFVDKEPRQTHGIWQAILHSDSIYFEVAAGVTVFVLAGRFFEARAKSKAG 234
CtpB-Mcanet    ALRNARHRTTSMETLISVGIVAATAWSLSSVFGDQPPREGSGIWRAILNSDSIYLEVAAGVTVFVLAGRYFEARAKSKAG 235
CtpB-H37Rv     ALRNARHRTTSMETLISVGIVAATAWSLSSVFGDQPPREGSGIWRAILNSDSIYLEVAAGVTVFVLAGRYFEARAKSKAG 235
CtpB-CDC1551   ALRNARHRTTSMETLISVGIVAATAWSLSSVFGDQPPREGSGIWRAILNSDSIYLEVAAGVTVFVLAGRYFEARAKSKAG 235
CtpB-Mbovis    ALRNARHRTTSMETLISVGIVAATAWSLSSVFGDQPPREGSGIWRAILNSDSIYLEVAAGVTVFVLAGRYFEARAKSKAG 235
CtpB-Mmicro    ALRNARHRTTSMETLISVGIVAATAWSLSSVFGDQPPREGSGIWRAILNSDSIYLEVAAGVTVFVLAGRYFEARAKSKAG 235
CtpB-Erdman    ALRNARHRTTSMETLISVGIVAATAWSLSSVFGDQPPREGSGIWRAILNSDSIYLEVAAGVTVFVLAGRYFEARAKSKAG 235
CtpB-Mafrica   ALRNARHRTTSMETLISVGIVAATAWSLSSVFGDQPPREGSGIWRAILNSDSIYLEVAAGVTVFVLAGRYFEARAKSKAG 235
CtpB-Mmarin    AIRNARHRTASMETLISVGITAATVWSLSTVFVDRQPRHSRGIWQAILNSDSIYLEVAAGVTVFVLAGRYFEARAKSKAG 232
CtpB-Mulcer    AIRNARHRTASMETLISVGITAATVWSLSTVFVDRQPRQSRGIWQAILNSDSIYLEVAAGVTVFVLAGRYFEARAKSKAG 232
CtpA-Mlepra    AIHNARYHGASMETLISTGITAATIWSLYTVFGHHQSTEHRGVWRALLGSDAIYFEVAAGITVFVLAGKYYTARAKSHAS 205
CtpA-Mulcer    ALRNARHHGASMETLISVGITAATIWSLYTVFGSHQLADRKGIWQALLGSDAIYFEVAAGVTVFVLAGRYFEARAKSKAG 237
CtpA-MtbF11    AMRNARHHAASMETLISVGITAATIWSLYTVFGNHSPIERSGIWQALLGSDAIYFEVAAGVTVFVLVGRYFEARAKSQAG 232
CtpA-H37Rv     AMRNARHHAASMETLISVGITAATIWSLYTVFGNHSPIERSGIWQALLGSDAIYFEVAAGVTVFVLVGRYFEARAKSQAG 232

CtpB-Mlepra    SALRALAARGAKNVEVLLPNGAELTIPAGELKKQQHFLVRPGETITADGVVIDGTATIDMSAITGEARPVHASPASTVVG 314
CtpB-Mcanet    SALRALAELGAKNVAVLLPDGAELVIPASELKKRQRFVTRPGETIAADGVVVDGSAAIDMSAMTGEAKPVRAYPAASVVG 315
CtpB-H37Rv     SALRALAELGAKNVAVLLPDGAELVIPASELKKRQRFVTRPGETIAADGVVVDGSAAIDMSAMTGEAKPVRAYPAASVVG 315
CtpB-CDC1551   SALRALAELGAKNVAVLLPDGAELVIPASELKKRQRFVTRPGETIAADGVVVDGSAAIDMSAMTGEAKPVRAYPAASVVG 315
CtpB-Mbovis    SALRALAELGAKNVAVLLPDGAELVIPASELKKRQRFVTRPGETIAADGVVVDGSAAIDMSAMTGEAKPVRAYPAASVVG 315
CtpB-Mmicro    SALRALAELGAKNVAVLLPDGAELVIPASELKKRQRFVTRPGETIAADGVVVDGSAAIDMSAMTGEAKPVRAYPAASVVG 315
CtpB-Erdman    SALRALAELGAKNVAVLLPDGAELVIPASELKKRQRFVTRPGETIAADGVVVDGSAAIDMSAMTGEAKPVRAYPAASVVG 315
CtpB-Mafrica   SALRALAELGAKNVAVLLPDGAELVIPASELKKRQRFVTRPGETIAADGVVVDGSAAIDMSAMTGEAKPVRAYLAASVVG 315
CtpB-Mmarin    GALRALAALGAKDVTVLLPDGAELVIPAAELKKRQRFVTRPGQTIAADGVVIDGTAAIDMSAMTGESKPVRATADAPVIG 312
CtpB-Mulcer    GALRALAALGAKDVTVLLPDGAELVIPAAELKKRQRFVTRPGQTIAADGVVIDGTAAIDMSAMTGESKPVRATADAPVIG 312
CtpA-Mlepra    IALLALAALSAKDAAVLQPDGSEMVIPANELNEQQRFVVRPGQTIAADGLVIDGSATVSMSPITGEAKPVRVNPGAQVIG 285
CtpA-Mulcer    AALRALAALSAKEVAVLQPDGSELVVPADELQEQDRFVVRPGQTIAADGLVLDGSAAVDMSTMTGEAKPTRVRPGGQVIG 317
CtpA-MtbF11    SALRALAALSAKEVAVLLPDGSEMVIPADELKEQQRFVVRPGQIVAADGLAVDGSAAVDMSAMTGEAKPTRVRPGGQVIG 312
CtpA-H37Rv     SALRALAALSAKEVAVLLPDGSEMVIPADELKEQQRFVVRPGQIVAADGLAVDGSAAVDMSAMTGEAKPTRVRPGGQVIG 312

CtpB-Mlepra    GTTVLDGRLVIEATAVGGDTQFAAMVRLVEDAQVQKARVQHLADRIAAVFVPMVFVIAGLAGASWLLAGASPDRAFSVVL 394
CtpB-Mcanet    GTVVMDGRLVIEATAVGADTQFAAMVRLVEQAQTQKARAQRLADHIAGVFVPVVFVIAGLAGAAWLVSGAGADRAFSVTL 395
CtpB-H37Rv     GTVVMDGRLVIEATAVGADTQFAAMVRLVEQAQTQKARAQRLADHIAGVFVPVVFVIAGLAGAAWLVSGAGADRAFSVTL 395
CtpB-CDC1551   GTVVMDGRLVIEATAVGADTQFAAMVRLVEQAQTQKARAQRLADHIAGVFVPVVFVIAGLAGAAWLVSGAGADRAFSVTL 395
CtpB-Mbovis    GTVVMDGRLVIEATAVGADTQFAAMVRLVEQAQTQKARAQRLADHIAGVFVPVVFVIAGLAGAAWLVSGAGADRAFSVTL 395
CtpB-Mmicro    GTVVMDGRLVIEATAVGADTQFAAMVRLVEQAQTQKARAQRLADHIAGVFVPVVFVIAGLAGAAWLVSGAGADRAFSVTL 395
CtpB-Erdman    GTVVMDGRLVIEATAVGADTQFAAMVRLVEQAQTQKARAQRLADHIAGVFVPVVFVIAGLAGAAWLVSGAGADRAFSVTL 395
CtpB-Mafrica   GTVVMDGRLVIEATAVGADTQFAAMVRLVEQAQTQKARAQRLADHIAGVFVPVVFVIAGLAGAAWLVSGAGADRAFSVTL 395
CtpB-Mmarin    GTIVLDGRLVIEATAVGADTQFAAMVRLVEEAQAQKARAQRLADRISAVFVPVVFAIAGLAGAAWLLSGAGADRAFSVTL 392
CtpB-Mulcer    GTIVLDGRLVIEATAVGADTQFAAMVRLVEEAQAQKARAQRLADRISAVFVPVVFAIAGVAGVAWMLSGAGADRAFSVTL 392
CtpA-Mlepra    GTVVLNGRLIVEAAAVGDETQLAGMVRLVEQAQQQNANAQRLADRIASVFVPCVFAVAALTAVGWLIAGSGPDRVFSAAI 365
CtpA-Mulcer    GTVVLDGRLIVEAAAVGADTRFAGMVRLVEQAQAQKADAQRLADRIASVFVPCVFVIAALTAAGWLLAGGSPDRAFSAAI 397
CtpA-MtbF11    GTTVLDGRLIVEAAAVGADTQFAGMVRLVEQAQAQKADAQRLADRISSVFVPAVLVIAALTAAGWLIAGGQPDRAVSAAL 392
CtpA-H37Rv     GTTVLDGRLIVEAAAVGADTQFAGMVRLVEQAQAQKADAQRLADRISSVFVPAVLVIAALTAAGWLIAGGQPDRAVSAAL 392
```

```
CtpB-Mlepra  GVLVIACPCTLGLATPTAMMVASGRGAQLGIFIKGYRALETINAIDTVVFDKTGTLTLGQLSVSTVTSTGG----WCSGE 470
CtpB-Mcanet  GVLVIACPCALGLATPTAMMVASGRGAQLGIFIKGYRALETIRSIDTVVFDKTGTLTVGQLAVSTVTMAGSGTSERDREE 475
CtpB-H37Rv   GVLVIACPCALGLATPTAMMVASGRGAQLGIFIKGYRALETIRSIDTVVFDKTGTLTVGQLAVSTVTMAGSGTSERDREE 475
CtpB-CDC1551 GVLVIACPCALGLATPTAMMVASGRGAQLGIFIKGYRALETIRSIDTVVFDKTGTLTVGQLAVSTVTMAGSGTSERDREE 475
CtpB-Mbovis  GVLVIACPCALGLATPTAMMVASGRGAQLGIFIKGYRALETIRSIDTVVFDKTGTLTVGQLAVSTVTMAGSGTSERDREE 475
CtpB-Mmicro  GVLVIACPCALGLATPTAMMVASGRGAQLGIFIKGYRALETIRSIDTVVFDKTGTLTVGQLAVSTVTMAGSGTSERDREE 475
CtpB-Erdman  GVLVIACPCALGLATPTAMMVASGRGAQLGIFIKGYRALETIRSIDTVVFDKTGTLTVGQLAVSTVTMAGSGTSERDREE 475
CtpB-Mafrica GVLVIACPCALGLATPTAMMVASGRGAQLGIFIKGYRALETIRSIDTVVFDKTGTLTVGQLAVSTVTMAGSGTSERDREE 475
CtpB-Mmarin  GVLVIACPCALGLATPTAMMVASGRGAQLGIFIKGYQALETIHGIDAVVFDKTGTLTVGKLTVNTVTTTGG----RVREE 468
CtpB-Mulcer  GVLVIACPCALGLATPTAMMVASGRGAQLGIFIKGYQALETIHGIDAVVFEKTGTLTVGKLTVNTVTTTGG----RVREE 468
CtpA-Mlepra  AVLVIACPCALGLATPTAMMVASGRGAQLGILLKGHESFEATRAVDTVVFDKTGTLTTGQLKVSAVTAAPG----WQANE 441
CtpA-Mulcer  AVLVIACPCALGLATPTAMMVASGRGAQLGIFLKGYQSLEATRSVDTVVFDKTGTLTSGQLKVSAVVAAPG----WDADE 473
CtpA-MtbF11  AVLVIACPCALGLATPTAMMVASGRGAQLGIFLKGYKSLEATRAVDTVVFDKTGTLTTGRLQVSAVTAAPG----WEADQ 468
CtpA-H37Rv   AVLVIACPCALGLATPTAMMVASGRGAQLGIFLKGYKSLEATRAVDTVVFDKTGTLTTGRLQVSAVTAAPG----WEADQ 468

CtpB-Mlepra  VLALASAVEAASEHSVATAIVAAYADPRPVADFVAFAGCGVSGVVAEHHVKIGKPSWVTRNAPC-DVVLESARREGESRG 549
CtpB-Mcanet  VLGLAAAVESASEHAMAAAIVAASPDPGPVNGFVAVAGCGVSGEVGGHHVEVGKPTWITRTTPCHDAALVSARLDGESRG 555
CtpB-H37Rv   VLGLAAAVESASEHAMAAAIVAASPDPGPVNGFVAVAGCGVSGEVGGHHVEVGKPSWITRTTPCHDAALVSARLDGESRG 555
CtpB-CDC1551 VLGLAAAVESASEHAMAAAIVAASPDPGPVNGFVAVAGCGVSGEVGGHHVEVGKPSWITRTTPCHDAALVSARLDGESRG 555
CtpB-Mbovis  VLGLAAAVESASEHAMAAAIVAASPDPGPVNGFVAVAGCGVSGEVGGHHVEVGKPSWITRTTPCHDAALVSARLDGESRG 555
CtpB-Mmicro  VLGLAAAVESASEHAMAAAIVAASPDPGPVNGFVAVAGCGVSGEVGGHHVEVGKPSWITRTTPCHDAALVSARLDGESRG 555
CtpB-Erdman  VLGLAAAVESASEHAMAAAIVAASPDPGPVNGFVAVAGCGVSGEVGGHHVEVGKPSWITRTTPCHDAALVSARLDGESRG 555
CtpB-Mafrica VLGLAAAVESASEHAMAAAIVAASPDPGPVNGFVAVAGCGVSGEVGGHHVEVGKPSWITRTTPCHDAALVSARLDGESRG 555
CtpB-Mmarin  VLALASAVEAASEHAVATAIVAASPHPAPVTDFVAVAGCGVSGIVDGHRIEVGKPSWITRNTAA-DEVLDTARAQGESRG 547
CtpB-Mulcer  VLALASAVEAASEHAVATAIVAASPHPAPVTDFVAVAGCGVSRIVDGHRIEVGKPSWITRNTAA-DEVLDTARAQGESRG 547
CtpA-Mlepra  VLQMAATVESASEHAVALAIAASTTHREPVANFRAVPGHGVSGTVAERAVRVGKPSWI--ASRCNSTTLVTARRNAELRG 519
CtpA-Mulcer  VLAIAATVEAASEHSVALAIAAATTQHDTVTEFRAIPGRGVSGTVSGRSVRVGKPSWIG-SSSS-DTALRAALRNAESLG 551
CtpA-MtbF11  VLALAATVEAASEHSVALAIAAATTRRDAVTDFRAIPGRGVSGTVSGRAVRVGKPSWIG-SSSC-HPNMSAARRHAESLG 546
CtpA-H37Rv   VLALAATVEAASEHSVALAIAAATTRRDAVTDFRAIPGRGVSGTVSGRAVRVGKPSWIG-SSSC-HPNMRAARRHAESLG 546

CtpB-Mlepra  ETVVFVSVDGVACGAVAIADTVKDSAADAISALCSRGLHTILLTGDNQAAARAVAAQVGIDTVIADMLPEAKVDVIQRLR 629
CtpB-Mcanet  ETVVFVSVDGVVRAALTIADTLKDSAAAAVAALRSRGLRTILLTGDNRAAADAVAAQVGIDSAVADMLPEGKVDVIQRLR 635
CtpB-H37Rv   ETVVFVSVDGVVRAALTIADTLKDSAAAAVAALRSRGLRTILLTGDNRAAADAVAAQVGIDSAVADMLPEGKVDVIQRLR 635
CtpB-CDC1551 ETVVFVSVDGVVRAALTIADTLKDSAAAAVAALRSRGLRTILLTGDNRAAADAVAAQVGIDSAVADMLPEGKVDVIQRLR 635
CtpB-Mbovis  ETVVFVSVDGVVRAALTIADTLKDSAAAAVAALRSRGLRTILLTGDNRAAADAVAAQVGIDSAVADMLPEGKVDVIQRLR 635
CtpB-Mmicro  ETVVFVSVDGVVRAALTIADTLKDSAAAAVAALRSRGLRTILLTGDNRAAADAVAAQVGIDSAVADMLPEGKVDVIQRLR 635
CtpB-Erdman  ETVVFVSVDGVVRAALTIADTLKDSAAAAVAALRSRGLRTILLTGDNRAAADAVAAQVGIDSAVADMLPEGKVDVIQRLR 635
CtpB-Mafrica ETVVFVSVDGVVRAALTIADTLKDSAAAAVAALRSRGLRTILLTGDNRAAADAVAAQVGIDSAVADMLPEGKVDVIQRLR 635
CtpB-Mmarin  ETIVFVSVDGEVCAAVTISDTVKDSAADAIAALRSRGLRTILLTGDNQAAADSVATQLGIDTAIADVLPDGKVNVIRQLR 627
CtpB-Mulcer  ETIVFVSVDGEVCAAVTISDTVKDSAADAIAALRSRGLRTILLTGDNQAVADSVATQLGIDTAIADVLPDGKVNVIRQLR 627
CtpA-Mlepra  ETAVFVEIDGEQCGVIAVADAVKASAADAVAALHDRGFRTALLTGDNPASAAAVASRIGIDEVIADILPEDKVDVIEQLR 599
CtpA-Mulcer  ETAVFVEIDGGPCGVIAVADAVKDSAADAVAALRERGLRTMLLTGDNPASAAAVADRVGIDEVIANILPEGKVDVIEQLR 631
CtpA-MtbF11  ETAVFVEVDGEPCGVIAVADAVKDSARDAVAALADRGLRTMLLTGDNPESAAAVATRVGIDEVIADILPEGKVDVIEQLR 626
CtpA-H37Rv   ETAVFVEVDGEPCGVIAVADAVKDSARDAVAALADRGLRTMLLTGDNPESAAAVATRVGIDEVIADILPEGKVDVIEQLR 626
```

```
CtpB-Mlepra   DQGHTVAMVGDGINDGPALACADLGLAMGRGTDVAIGAADLILVRDSLGVVPVALDLARATMRTIRINMIWAFGYNVAAI 709
CtpB-Mcanet   EEGHTVAMVGDGINDGPALVGADLGLAIGRGTDVALGAADIILVRDDLNTVPQALDLARATMRTIRMNMIWAFGYNVAAI 715
CtpB-H37Rv    EEGHTVAMVGDGINDGPALVGADLGLAIGRGTDVALGAADIILVRDDLNTVPQALDLARATMRTIRMNMIWAFGYNVAAI 715
CtpB-CDC1551  EEGHTVAMVGDGINDGPALVGADLGLAIGRGTDVALGAADIILVRDDLNTVPQALDLARATMRTIRMNMIWAFGYNVAAI 715
CtpB-Mbovis   EEGHTVAMVGDGINDGPALVGADLGLAIGRGTDVALGAADIILVRDDLNTVPQALDLARATMRTIRMNMIWAFGYNVAAI 715
CtpB-Mmicro   EEGHTVAMVGDGINDGPALVGADLGLAIGRGTDVALGAADIILVRDDLNTVPQALDLARATMRTIRMNMIWAFGYNVAAI 715
CtpB-Erdman   EEGHTVAMVGDGINDGPALVGADLGLAIGRGTDVALGAADIILVRDDLNTVPQALDLARATMRTIRMNMIWAFGYNVAAI 715
CtpB-Mafrica  EEGHTVAMVGDGINDGPALVGADLGLAIGRGTDVALGAADIILVRDDLNTVPQALDLARATMRTIRMNMIWAFGYNVAAI 715
CtpB-Mmarin   DQGHTVAMVGDGINDGPALVSADLGLAIGRGTDVAIGAADIILVRDDLHIVGQALDLARATLRTIRTNMIWAFGYNVAAI 707
CtpB-Mulcer   DQGHTVAMVGDGINDGPALVSADLGLAIGRGTDVAIGAADIILVRDDLHIVGQALDLARATMRTIRTNMIWAFGYNVAAI 707
CtpA-Mlepra   DRGHVVAMVGDGINDGPALARADLGMAIGRGTDVAIGAADIILVRDNLDVVPITLDLAAATMRTIKFNMVWAFGYNIAAI 679
CtpA-Mulcer   DRGHVVAMVGDGINDGPALASADLGMAMGRGTDVAIGAADIILVRDNLDVVPLALGLAAATMRTVKLNLAWAFGYNIAAI 711
CtpA-MtbF11   DRGHVVAMVGDGINDGPALARADLGMAIGRGTDVAIGAADIILVRDHLDVVPLALDLARATMRTVKLNMVWAFGYNIAAI 706
CtpA-H37Rv    DRGHVVAMVGDGINDGPALARADLGMAIGRGTDVAIGAADIILVRDHLDVVPLALDLARATMRTVKLNMVWAFGYNIAAI 706

CtpB-Mlepra   PIASSGLLNPLIAGAAMAFSSFFVVSNSLRLSNFG--------------------------------------------- 744
CtpB-Mcanet   PIAAAGLLNPLIAGAAMAFSSFFVVSNSLRLRNFG--------------------------------------------- 750
CtpB-H37Rv    PIAAAGLLNPLIAGAAMAFSSFFVVSNSLRLRNFG--------------------------------------------- 750
CtpB-CDC1551  PIAAAGLLNPLIAGAAMAFSSFFVVSNSLRLRNFG--------------------------------------------- 750
CtpB-Mbovis   PIAAAGLLNPLIAGAAMAFSSFFVVSNSLRLRNFG--------------------------------------------- 750
CtpB-Mmicro   PIAAAGLLNPLIAGAAMAFSSFFVVSNSLRLRNFG--------------------------------------------- 750
CtpB-Erdman   PIAAAGLLNPLIAGAAMAFSSFFVVSNSLRLRNFG--------------------------------------------- 750
CtpB-Mafrica  PIAAAGLLNPLIAGAAMAFSSFFVVSNSLRLRNFG--------------------------------------------- 750
CtpB-Mmarin   PIAAAGLLNPLIAGAAMAFSSFFVVSNSLRLRNFG--------------------------------------------- 742
CtpB-Mulcer   PIAAAGLLNPLIAGAAMAFSSFFVVSNSLRLRNFG--------------------------------------------- 742
CtpA-Mlepra   PIAAAGLLNPLVAGAAMAFSSFFVVSNSLRLRNFG-AILSCGTSRHRTVKRWRCPPPTRLRSTACSPVDASPLRPVAHRT 758
CtpA-Mulcer   PIAAAGVLNPVVAGAAMAFSSFFVVSNSLQLRNFGAATSQTTT-------------------------------------S 755
CtpA-MtbF11   PVAAAGLLNPLVAGAAMAFSSFFVVSNSLRLRKFGRYPLGCGT---------------------------------VGGP 753
CtpA-H37Rv    PVAAAGLLNPLVAGAAMAFSSFFVVSNSLRLRKFGRYPLGCGT---------------------------------VGGP 753

CtpB-Mlepra   --LSQTSD---------- 750
CtpB-Mcanet   ------AQ---------- 752
CtpB-H37Rv    ------AQ---------- 752
CtpB-CDC1551  ------AQ---------- 752
CtpB-Mbovis   ------AQ---------- 752
CtpB-Mmicro   ------AQ---------- 752
CtpB-Erdman   ------AQ---------- 752
CtpB-Mafrica  ------AQ---------- 752
CtpB-Mmarin   ---PPTARIVASNGEIDD 757
CtpB-Mulcer   ---PPTARIVASNGEIDD 757
CtpA-Mlepra   GVKPPTHR---------- 766
CtpA-Mulcer   TTEPGATE---------- 763
CtpA-MtbF11   QMTAPSSA---------- 761
CtpA-H37Rv    QMTAPSSA---------- 761
```

**Table S1. Plasmids**		
**Plasmid**	**Relevant features**	**Source/Reference**
pGH542	*oriM*, *oriE*, Tet^R, Tn1000 γδ-resolvase gene *tnpR* under control of mycobacterial Hsp60 promoter	DOI:10.1007/978-1-59745-232-8_15
pJV53	Kan^R, *oriE*, *oriM*, mycobacteriophage Che9c gp60/gp61 controlled from an acetamide-inducible promoter	DOI:10.1007/978-1-59745-232-8_15
pMV306	*oriE,aph,* mycobacteriophage L5 *att*/*int*	DOI:10.1128/iai.63.3.799-803.1995
pOSN5	pMV306 containing *M. tuberculosis ctpB* with upstream regulatory sequences	This study
pOSN19	pOSN20 after removal of *sigC*	This study
pOSN20	pSR173 with *lacZ* promoter cloned upstream of P_{tb21} to enhance expression *tetR(B)*	This study
pOSN21	pYUB854 with ~1-kb regions upstream and downstream of *M. tuberculosis ctpB* gene flanking the Hyg^R cassette	This study
pOSN26	pOSN20 with *M. tuberculosis ctpB* downstream of the $P_{myctetO}$ promoter	This study
pOSN30	pOSN20 encoding *M. tuberculosis ctpV* downstream of the $P_{myctetO}$ promoter	This study
pOSN37	pOSN20 encoding c-myc tag fusion to *M. tuberculosis ctpB* downstream of the $P_{myctetO}$ promoter	This study
pOSN38	pOSN20 encoding c-myc tag fusion to *M. tuberculosis ctpV* downstream of the $P_{myctetO}$ promoter	This study
pOSN40	pOSN5 with the kanamycin-resistance gene replaced with a gentamycin-resistance gene	This study
pSR173	*oriE*, *oriM*, *tetR(B)*, *hygR*, $P_{myctetO}$-*sigC*	DOI:10.1128/JB.01371-06
pYUB854	cos, *oriE*, *hyg* flanked by γδ-resolvase sites	DOI:10.1016/s0076-6879(02)58081-2

**Table S2. Bacterial strains**		
**Strain ID**	**Relevant features**	**Reference/source**
TAM1	*E. coli: mcrA, Δ(mrr-hsdRMS-mcrBC), Φ80lacZΔM15, ΔlacX74, recA1, araΔ139, (ara-leu)7697, galU, galK, rpsL, endA1, nupG*	Active Motif, Inc.
GA1505	TAM1/pOSN5	This study
GA1530	TAM1/pOSN21	This study
GA1568	TAM1/pOSN40	This study
GA1528	TAM1/pOSN19	This study
GA1509	TAM1/pOSN20	This study
GA1565	TAM1/pOSN37	This study
GA1566	TAM1/pOSN38	This study
mc^2155	*M. smegmatis* wildtype strain	DOI:10.1111/j.1365-2958.1990.tb02040.x
BCG	*M.. bovis* bacille Calmette-Guérin Pasteur	Sakamoto laboratory
BD3	mc^2155/pOSN19	This study
BD4	mc^2155/pOSN20	This study
BD5	mc^2155/pOSN26	This study
BD6	mc^2155/pOSN29	This study
BD7	mc^2155/pOSN30	This study
BD9	mc^2155/pOSN37	This study
BD10	mc^2155/pOSN38	This study
BD43	BCG*ΔctpB*::*hyg*	This study
BD63	mc^2 6206/pOSN19	This study
BD65	mc^2 6206/pOSN37	This study
BD66	mc^2 6206/pOSN38	This study
Erdman	*M. tuberculosis* strain Erdman	TB Branch, CDC
TB716	*M. tuberculosisΔctpB*::*hyg*	This study
TB717	*M. tuberculosisΔctpB::hyg*/pOSN40	This study
M.tbΔsigC	*M. tuberculosisΔsigC*	DOI:10.3390/ijms22042118
TB722	*M. tuberculosisΔsigC/*pOSN19	This study
TB723	*M. tuberculosisΔsigC/*pOSN20	This study
TB724	*M. tuberculosisΔsigC/*pOSN36	This study
TB725	*M. tuberculosisΔsigC/*pOSN37	This study
TB726	*M. tuberculosisΔsigC/*pOSN38	This study

Table S3. Primers	
**Primer**	**Sequence 5´ to 3´**
P1196	AGTGAGCTCTACGCCATCCC
P1575	GTGAAGCTTGTCAAGG TTGCGGTCGC GAC
P1576	GTGAAGCTTGCGGTCCATCGACGGAAAATTGC
P1629	CAA CAG CGA CTC GAT CTA CCT CG
P1630	G GAC CGT CGT TGA TGC CGT C
P1631	GGT TAT CAA GTG AGA AAT CAC CAT GAG TGA CG
P1703	CACGTG CAC CCC AGG CTT TAC ACT TTA TGC TTC CGG CTC GTA TGT TGT GTG GA GTTAAC
P1704	GTTAAC TC CAC ACA ACA TAC GAG CCG GAA GCA TAA AGT GTA AAG CCT GGG GTG CACGTG
P1705	CACGTG CTT TAC ACT TTA TGC TTC CGG CTC GTA TGT TGT GTG GAA TTG TGA GCG GAT AAC AAT TTC GTTAAC
P1706	GTTAAC GAA ATT GTT ATC CGC TCA CAA TTC CAC ACA ACA TAC GAG CCG GAA GCA TAA AGT GTA AAG CACGTG
P1896	AA GGA AGC CAC GCT GCT TGT CGA CG
P1897	TCT CCG ACT TGG ACC TGA TCG ACC
P2040	A CTC TGG GCT GG GTG GAG CAG AAG CTG ATC TCG GAA GAG GAC CTG GCG GCT CCA GTT GTG GGA GAT GCC
P2041	CCAATGCATCTA TTG AGC GCC AAA ATT GCG TAG CCG CAA GC
P2042	CACTACTTAAGCTCGCGATCGGACGAAGA
P2043	GCTCTAGACACGGCCCTCCTCGGATC
P2044	TTAGCCATGGGCTCAATAGCATGCCCAGC
P2045	TAGAAGATCTTGATCGCCTGGCTGACTGG
P2250	GTGTGCTAGCCCCGG GAA TTA ATT CTG CTC GCG CAG
P2251	GGACTAGT GGC CGC GGC GTT GTG AC
P2057	GAGGTTAATTAAGGTGA CGA CGG CCG TGA CCG
P2058	TGACATGCAT TC ACG CGG ACG ACG GCG C
P2059	GAGGTTAATTAAG GTG GCG GCT CCA GTT GTG GG
P2060	GAGGTTAATTAAGGT GCG CGT GTG CGT CAC CGG
P2061	TGACATGCATT CAT GCG GTT CGG CCG TCG C
